# Paired neutrophil association and ex vivo perturbation sensitivity define a bounded myeloid activation model in ARDS

**DOI:** 10.1371/journal.pone.0362747

**Published:** 2026-11-12

**Authors:** Wei Wei, Jing Liu, Ying Zhou, Rui Zhao, LinGang Zhang

**Affiliations:** 1 Emergency Department, Yuncheng Central Hospital, Yuncheng, Shanxi, China; 2 Pathology Department, Yuncheng Central Hospital, Yuncheng, Shanxi, China; Versiti Blood Research Institute, UNITED STATES OF AMERICA

## Abstract

**Background:**

Acute respiratory distress syndrome (ARDS) is biologically heterogeneous, but how myeloid transcriptional programmes relate across blood and lung compartments remains uncertain. We examined whether a pre-specified neutrophil effector/degranulation programme and a separately derived perturbation-response module provide convergent, boundary-aware evidence across public cohorts.

**Methods:**

Patient-level analyses descriptively localized programmes in a seven-patient peripheral blood mononuclear cell cohort and assessed paired bronchoalveolar lavage fluid and longitudinal alveolar-macrophage profiles. PBMC disease groups were severely confounded with expression-inferred sex. In purified neutrophils, paired ARDS–healthy-volunteer comparisons and healthy-donor granulocyte–macrophage colony-stimulating factor (GM-CSF)/broad phosphoinositide 3-kinase (PI3K) perturbations were analysed separately. A donor-adjusted whole-transcriptome weighted gene coexpression network analysis (WGCNA) derived a module under frozen retention rules, which was externally projected using fixed loadings and a pre-specified coverage gate.

**Results:**

The neutrophil-degranulation programme was higher in all 12 ARDS–healthy-volunteer pairs (mean paired difference, 0.0321; Benjamini–Hochberg false-discovery rate [BH-FDR] = 0.000977), increased after GM-CSF in 9 of 10 donors (BH-FDR = 0.0276) and decreased after broad pan-PI3K inhibition in all 10 donors (BH-FDR = 0.00469). No gene passed the genome-wide three-direction conjunction threshold; SERPINB1, PNP and TLR2 were retained only as pathway-restricted directional response anchors. A separate 627-gene green module met the perturbation-response rule (conjunction BH-FDR = 3.50 × 10⁻⁴) and showed functional convergence with limited gene-level overlap. PBMC localization was descriptive, without an interpretable ARDS–sepsis contrast. External green projection showed post hoc monocyte/macrophage-biased ordering rather than neutrophil specificity. In eight paired BALF patients, the pre-specified contrast was directionally opposite but inconclusive (P = 0.571); longitudinal outcome evidence was inconsistent.

**Conclusions:**

These data support an ARDS-associated myeloid/neutrophil effector programme with paired disease association and ex vivo GM-CSF/pan-PI3K sensitivity. They support mechanistic prioritization of compartment-dependent myeloid biology, but not a prognostic signature, a causal programme-to-outcome mechanism, PI3K isoform specificity or PI3K-directed therapeutic efficacy.

## Introduction

Acute respiratory distress syndrome (ARDS) is a common form of acute hypoxaemic respiratory failure that arises from diverse direct and indirect insults and remains associated with substantial mortality and long-term morbidity. Its clinical syndrome is defined by impaired alveolar–capillary barrier function, but the underlying host response is neither uniform across patients nor confined to a single biological compartment [1–3]. This heterogeneity has complicated efforts to relate circulating molecular measurements to pulmonary injury, to distinguish inflammatory responses that accompany disease from those that shape its course, and to identify mechanistically credible targets for prospective testing.

Myeloid cells and neutrophils are plausible contributors to this heterogeneity. In ARDS, circulating neutrophil protease–inhibitor imbalance has been associated with disease onset, consistent with altered effector activation during early injury [4–6]. However, a transcriptional programme measured in one compartment cannot be assumed to represent the same cell state or biological function in another. Peripheral-blood monocytes and alveolar macrophages have distinct transcriptional programmes in ARDS [7], and longitudinal CITE-seq profiling of acute hypoxaemic respiratory failure has resolved dynamic alveolar monocyte/macrophage subsets with divergent inflammatory and reparative features [8]. These observations motivate an explicitly compartment-aware approach to interpreting myeloid transcriptional signals.

Public transcriptomic cohorts offer complementary opportunities to examine this biology, but they also introduce interpretive risks. Biological ARDS subphenotypes have been associated with distinct outcomes and treatment-response patterns, reinforcing the need to preserve cohort and design boundaries [9,10]. Datasets differ in sampled compartment, disease aetiology, cell enrichment, longitudinal endpoint and platform coverage. Single-cell studies can localize programmes to cellular states, yet inferential units remain patients rather than cells; patient-level aggregation limits the false discoveries that arise when cells are treated as independent replicates [11–13]. Similarly, related analyses from the same accession may provide within-study support but cannot be counted as independent replication. Consequently, a robust cross-cohort analysis should preserve matched-pair and patient-level structure, freeze gene-set and module definitions before external evaluation, and retain negative and platform-limited findings alongside positive results.

Here, we organized public ARDS cohorts by biological compartment and evidentiary role to examine an ARDS-associated myeloid/neutrophil effector programme. We first assessed pre-specified programmes across patient-level PBMC, paired bronchoalveolar lavage fluid and longitudinal alveolar-macrophage analyses. We then used purified neutrophils to test paired ARDS association and ex vivo GM-CSF induction with broad pan-PI3K inhibitor sensitivity. In parallel, we derived a donor-adjusted whole-transcriptome coexpression module from the perturbation experiment, while keeping it analytically distinct from pathway-restricted directional response anchors. Finally, we projected frozen module loadings into external cohorts to examine cellular localization and longitudinal boundaries without refitting. This design was intended to distinguish disease association and ex vivo perturbation sensitivity from unsupported claims about cellular specificity, prognosis, causality or therapeutic efficacy.

## Materials and methods

### Study design and analytical framework

We performed a secondary analysis of publicly available human transcriptomic datasets spanning peripheral blood, bronchoalveolar lavage fluid (BALF), alveolar macrophages and purified neutrophils. Cohorts were assigned complementary roles according to biological compartment, study design and available outcomes. They were processed independently, and expression matrices were never merged across platforms.

The primary analysis comprised two complementary routes. A hypothesis-driven route projected four externally defined transcriptional programmes and tested directional convergence within a pre-specified neutrophil-degranulation programme. A data-driven route constructed a donor-adjusted coexpression network from the GSE76293 perturbation experiment. The two routes shared GSE76293 and were therefore not treated as independent biological validation. Their outputs were kept separate at gene level and compared only through frozen overlap and functional-enrichment analyses.

All analyses used the patient, matched pair or experimental donor as the biological unit. Cell-level embeddings were used for annotation and visualization, whereas single-cell inference used patient-level pseudobulk profiles. Membership rules, directional hypotheses, multiplicity families and external-projection gates were fixed before the corresponding results were inspected whenever indicated below. Negative and coverage-limited results were retained without outcome-driven threshold relaxation.

### Public datasets and cohort roles

We used Gene Expression Omnibus accessions GSE151263, GSE240580, GSE116560, GSE76293, GSE273149, GSE89953, GSE32707 and GSE207077. GSE151263 contained peripheral-blood single-cell RNA sequencing data from three patients with ARDS and four patients with sepsis [14]. GSE240580 contained 16 paired BALF single-cell libraries from eight patients sampled at two time points, including five patients classified as resolved and three as not resolved at follow-up [15]. GSE116560 contained 68 longitudinal alveolar-macrophage microarrays from 35 patients on Days 1, 4 and 8, with 28-day ventilator-free days (VFD) as the outcome [16].

GSE76293 contained 94 purified-neutrophil microarrays in two experimental parts [17]. Part A comprised 12 matched ARDS and healthy-volunteer pairs. Part B comprised ten healthy donors studied under seven conditions. Programme and gene-level perturbation analyses used the 6-h DMSO, GM-CSF and GM-CSF plus broad pan-PI3K conditions. Network discovery used all six 6-h treatment conditions and excluded the zero-hour fresh samples. GSE273149 contained 49 longitudinal PBMC RNA-sequencing profiles from four COVID-19 ARDS survivors, five COVID-19 ARDS non-survivors and four non-COVID ARDS patients sampled on nominal Days 1, 3, 7 and 10 [18].

Three datasets defined contextual boundaries. GSE89953 contained paired Day-1 alveolar-macrophage and peripheral-blood monocyte profiles from 26 patients with ARDS [7]. All 26 alveolar-macrophage arrays were byte-identical to corresponding GSE116560 Day-1 arrays and were not counted as independent evidence. GSE32707 provided Day-0 whole-blood profiles from ICU controls and patients with SIRS, sepsis or sepsis-associated ARDS [19]. GSE207077 provided transformed whole-BALF expression profiles from 19 patients with severe COVID-19 and nine patients with influenza-associated ARDS [20].

We audited treatment information in the source cohorts. The GSE240580 source supplementary table reported a daily maximum steroid dose for seven of eight patients and listed one as not applicable [15]. The GSE116560 source supplementary clinical record noted systemic steroid use at some point during the clinical course for six of 35 patients [16]. GSE116560 came from a randomized fish-oil trial; the source study reported a sensitivity analysis excluding recipients, but GEO sample annotations lacked treatment assignment [16]. In GSE273149, the source study reported dexamethasone for all four COVID-19 ARDS survivors, four of five COVID-19 ARDS non-survivors and none of four non-COVID ARDS controls [18]. GEO annotations for GSE76293 Part A did not contain patient medication fields. The GSE273149 study also reported remdesivir and convalescent plasma exposure by group. We did not add treatment covariates to the cohort-specific analyses because exposure timing and dose relative to each transcriptomic sample were incomplete, and treatment was nearly uniform within some small groups.

This work introduced no new patient recruitment or specimen collection. Analyses used de-identified public data and followed the access conditions of the source repositories.

### Pre-specified transcriptional programmes and rank scoring

Four programmes were frozen from MSigDB human release 2026.1 before outcome modelling [21]: HALLMARK_INFLAMMATORY_RESPONSE, REACTOME_NEUTROPHIL_DEGRANULATION, GOBP_APOPTOTIC_CELL_CLEARANCE and GOBP_WOUND_HEALING. Their frozen memberships contained 200, 478, 44 and 455 genes, respectively. Gene symbols were converted to uppercase and duplicate symbols were removed when the memberships were frozen. No cohort result was used to add, remove or reweight programme members.

Programme scores were calculated separately within each sample or pseudobulk profile. All measured genes were ranked using average ranks for ties and transformed to percentile ranks from 0 to 1. The score was the mean percentile rank of detected programme genes, mapped to the interval from −1 to –1 as

Score = 2 × (mean programme-gene percentile rank - 0.5).

At least 20 programme genes had to be detected. Programme coverage was recorded for every cohort. Rank scoring preserved within-profile ordering and avoided imposing a shared expression scale across microarray and RNA-sequencing platforms. Absolute scores were consequently interpreted only within cohorts and were not compared across platforms.

### GSE151263 single-cell processing and patient pseudobulks

The submitted matrices contained 24,806 pre-filtered cells from seven patients and 21,212 genes in a common order. Four spreadsheet-converted feature names were restored to their contemporaneous gene symbols using their invariant row positions across all seven matrices. Cells were required to express at least 200 genes and to contain no more than 20% mitochondrial UMIs. Doublets were identified independently within each sample using scDblFinder 1.26.7 with dbr.per1k = 0.008. After hard filtering and doublet removal, 23,485 core singlets were retained.

Counts were log-normalized to 10,000 transcripts per cell in Seurat [22]. We selected 3,000 variable features, scaled these features and calculated 50 principal components. Harmony correction used sample identity and the first 40 principal components for cell-identity alignment only [23]. A shared-nearest-neighbour graph used Harmony dimensions 1−30. Clusters were evaluated over resolutions 0.2–0.8, and the resolution-0.6 solution was frozen for annotation. Uniform manifold approximation and projection used the same 30 Harmony dimensions. Cluster identities were assigned from positive marker genes, canonical lineage markers, sample distribution and quality metrics, yielding 14 frozen annotations. Harmony-adjusted values were not used for programme scoring or differential-expression inference.

Raw UMIs were summed within each patient and frozen cell state. Profiles required at least 50 cells and 50,000 total UMIs. Programme localization used eligible patient pseudobulks. The three primary myeloid states represented in all seven patients were CD14 inflammatory monocytes, FCGR3A non-classical monocytes and low-density neutrophils. The 12 archived ARDS–sepsis Wilcoxon comparisons (four programmes × three states; Benjamini–Hochberg adjustment [24]) remain in source data but are not interpreted as disease tests. All three ARDS patients and one of four sepsis patients had a female expression-inferred sex proxy, not independently verified demographic sex.

For the separate exploratory pseudobulk expression audit, raw counts were analysed with edgeR quasi-likelihood models [25]. Genes were retained with filterByExpr, using minimum count and total-count settings of 10 and 15. Models used robust quasi-likelihood estimation and treated patients as the statistical units. Each myeloid-state analysis was repeated after leaving out each patient. Cell-state proportions were evaluated using all 35 possible allocations of three ARDS labels among seven patients, followed by Benjamini-Hochberg correction across annotated states. These exploratory audits neither defined programme membership nor supported ARDS–sepsis inference.

### GSE240580 BALF processing and paired longitudinal analysis

GSE240580 comprised 103,995 cells from 16 10x libraries. Cells were required to contain at least 200 UMIs, express at least 100 genes and contain no more than 25% mitochondrial UMIs. scDblFinder was run separately for every library with dbr.per1k = 0.008. After hard filtering and doublet removal, 87,115 core singlets remained.

Counts were log-normalized to 10,000 transcripts per cell. We selected 4,000 variable features and removed mitochondrial, ribosomal and haemoglobin features from that variable-feature set. Fifty principal components were calculated. Harmony correction used library identity and principal components 1−40 for annotation and visualization only. The neighbour graph and UMAP used Harmony dimensions 1−35. Clustering was evaluated at resolutions 0.4, 0.8 and 1.2, and the resolution-0.8 solution containing 29 clusters was frozen. Annotations integrated positive markers, canonical lineage markers, patient distribution and cluster-level quality summaries.

Raw UMIs were summed within each patient, time point and frozen broad or fine cell state. Profiles required at least 50 cells and 50,000 UMIs. The primary endpoint was the within-patient change in the broad-neutrophil neutrophil-degranulation score, calculated as follow-up minus baseline. We compared these changes between the five resolved and three not-resolved patients using a two-sided exact Wilcoxon rank-sum test. This was the single pre-specified primary test and was not multiplicity-adjusted.

Planned sensitivities excluded patient-enriched clusters 10 and 13, examined COVID-19 and non-COVID-19 strata, and recalculated the contrast after omitting each patient. The exploratory baseline wound-healing analysis compared eight broad-myeloid cell-state-by-programme combinations and used Benjamini-Hochberg correction within each time point. Cell-composition contrasts were corrected within their predefined composition families. No secondary result was permitted to override the frozen primary endpoint.

### GSE116560 microarray processing and longitudinal mixed models

We analysed the official GPL6883 sample tables for 68 alveolar-macrophage profiles from 35 patients. The submitted signals had undergone lumi variance stabilization and quantile normalization and had been returned to the natural scale. We applied log2 transformation but no second between-array normalization. Probes were retained using the pre-specified detection-P filter and mapped through the official platform annotation. Probes with a single gene symbol were collapsed by the within-sample median, producing 11,139 genes.

Six arrays were flagged for review from detection rate, high-variance-probe correlations and relative-log-expression metrics. Their correlations remained 0.81–0.90, and four were Day-8 samples. The primary analysis retained all arrays to avoid outcome- or time-dependent exclusion. Sensitivity analyses excluded these six arrays.

Programme scores were modelled using maximum-likelihood linear mixed-effects models with patient-specific random intercepts. The primary model was score ~ categorical time × standardized continuous VFD. Benjamini-Hochberg correction was applied across the four programmes for the time-by-VFD term within each analysis track. Sensitivity tracks added standardized age and sex, excluded the six quality-control-review arrays, or applied both changes. Binary VFD = 0 versus VFD > 0 trajectories and Day-1 associations were descriptive and did not replace the continuous-VFD primary model.

### GSE76293 preprocessing and paired programme analyses

We read all 94 official GPL570 processed sample tables. The source values were already log-scale normalized expression measurements. Probes with unambiguous official gene symbols were retained and multiple probes were collapsed by their mean, yielding 21,653 unique gene symbols. Programme scoring used the frozen rank-based method.

Part A included 12 matched ARDS–healthy neutrophil pairs. Each programme was tested using a two-sided Wilcoxon signed-rank test with Benjamini–Hochberg correction across four programmes. Part B contrasts were computed within ten donors: GM-CSF versus 6-h DMSO, and GM-CSF plus broad pan-PI3K inhibitor versus GM-CSF. Two-sided Wilcoxon signed-rank tests were corrected across the predefined programme-by-contrast family. The source study used rhGM-CSF (1 ng/mL) and the pan-class I PI3K inhibitor ZSTK474 (10 μM) for the 6-h arrays [17]. It also used 10 μM ZSTK474 in separate 20-h survival experiments, but an array-specific rationale for selecting this concentration could not be verified. GEO identifies the 6-h vehicle as DMSO without stating its final percentage. The 0.1% DMSO reported for the 20-h survival assay cannot be assigned to the arrays. The retrieved study report and GEO records did not provide condition-matched 6-h viability or Annexin V/propidium iodide values; RNA integrity numbers indicate RNA quality, not cell viability. The inhibitor contrast was therefore interpreted as an ex vivo transcriptional response without isoform-specific, viability-independent or therapeutic inference.

### Directional gene-level conjunction and frozen GSE273149 test

The gene-level GSE76293 analysis tested three pre-specified positive directions: ARDS minus matched healthy volunteer, GM-CSF minus 6-h DMSO, and GM-CSF minus GM-CSF plus broad pan-PI3K inhibitor. Each contrast used a separate paired limma model containing matched-pair or donor indicators [26]. Empirical Bayes moderation used robust variance estimation. Two-sided moderated P values were converted to one-sided values only for the fixed positive directions.

For each gene, the conjunction P value was the maximum of the three one-sided P values, constituting an intersection-union test. Benjamini-Hochberg correction was applied across all 21,653 genes for the genome-wide tier. Strict membership required a paired standardized effect of at least 0.5 and at least 75% positive biological units in every contrast. The mean effect also had to remain positive after every leave-one-unit-out omission. A second, pathway-restricted tier applied the same rules and restricted multiplicity correction to the 446 detected genes in the previously frozen neutrophil-degranulation programme. A tier required at least three retained genes. The three genes retained in this restricted tier, SERPINB1, PNP and TLR2, were frozen as directional response anchors rather than a genome-wide module.

The external anchor score in GSE273149 was specified before expression access. Within each sample, positive-count genes were ranked and transformed to percentile ranks. The unweighted mean rank of SERPINB1, PNP and TLR2 was mapped to −1 to –1. All three genes were required, because two of three would fail the fixed 70% coverage threshold. We estimated a separate ordinary-least-squares score-on-day slope for each COVID-19 ARDS patient with at least three observations. The primary statistic was the difference between the mean slope in five non-survivors and four survivors. Its one-sided P value was exact over all 126 allocations of five non-survivor labels among nine patients. A supportive classification required a positive effect, P ≤ 0.05 and positive direction in at least 80% of leave-one-patient-out analyses. Non-COVID ARDS patients were displayed as etiological context and excluded from the primary test.

### Donor-adjusted WGCNA and frozen Part A projection

In brief, we adjusted donor and RNA-integrity effects while preserving treatment means, then grouped genes with correlated expression. We tested each module for the pre-specified GM-CSF increase and inhibitor reversal across donors, froze modules passing both rules, and only then examined the ARDS patient pairs and external cohorts.

Network discovery used the 60 GSE76293 Part B arrays representing ten donors under six paired 6-h conditions. Zero-hour samples and all Part A arrays were excluded from network construction and module selection. We used limma removeBatchEffect to remove additive donor and continuous RNA integrity number effects while preserving the six condition means. Genes required finite values and non-zero adjusted median absolute deviation. The upper 50% by adjusted median absolute deviation was retained without reference to treatment effects, leaving 10,827 genes.

We constructed a signed WGCNA network using biweight midcorrelation with maxPOutliers = 0.10 [27]. Candidate soft powers were 1–10 and the even powers 12–20. The smallest power with signed scale-free-fit R^2^ of at least 0.80 was selected. Network construction used deepSplit = 2, minModuleSize = 30, mergeCutHeight = 0.25, pamRespectsDendro = FALSE, reassignThreshold = 0, maxBlockSize = 6000 and random seed 20260811. Module eigengenes were oriented to correlate positively with mean standardized member-gene expression. Grey genes were treated as unassigned.

For every non-grey module and donor, we calculated the eigengene difference for GM-CSF minus vehicle and for GM-CSF minus GM-CSF plus broad pan-PI3K inhibitor. One-sided one-sample t tests evaluated the pre-specified positive direction. The module conjunction P value was the maximum of the two P values and was Benjamini-Hochberg adjusted across all non-grey modules. Strict modules required positive mean effects, paired standardized effects of at least 0.50 and at least 70% positive donors in both contrasts. Mean effects also had to remain positive in every leave-one-donor-out analysis, with conjunction BH-FDR ≤ 0.05.

Full membership of every strict module was frozen before Part A testing. Hubs required oriented module membership kME >= 0.70, positive mean effects and at least 70% positive donors in both Part B contrasts. Part A and external outcomes did not influence module or hub membership.

For each strict module, we derived PC1 loadings from standardized, donor-adjusted Part B expression. PC1 was oriented toward higher mean member-gene expression, and the Part B gene means, standard deviations and loadings were frozen. Part A arrays were projected without refitting. The one-sided paired Part A test was Benjamini-Hochberg adjusted across strict Part B modules. Support required a positive mean difference, paired standardized effect of at least 0.50, at least nine of 12 positive pairs, positive leave-one-pair-out means and BH-FDR ≤ 0.05. Adjacent-power module overlap was assessed without altering the selected network.

### Functional overlap and over-representation analysis

Functional analyses used the fixed 10,827-gene WGCNA universe. We compared the 627-member green module and its 86 frozen hubs with the 342 neutrophil-degranulation genes mapped to this universe. Enrichment was tested using one-sided Fisher exact tests, and the two P values were adjusted together using Benjamini-Hochberg correction. We reported exact odds ratios and 95% confidence intervals, Jaccard indices and overlap fractions. No inferential enrichment test was performed for the three anchors because they were selected within the same biological scope.

Over-representation analysis used frozen MSigDB 2026.1.Hs snapshots from Hallmark, Reactome and Gene Ontology Biological Process, obtained with msigdbr 26.1.0. Gene sets were restricted to 10–500 universe-mapped genes. One-sided hypergeometric tests were performed separately for the complete green module, frozen hubs and descriptive neutrophil-degranulation reference. Benjamini-Hochberg correction was applied across all eligible terms within each query-library combination.

For deterministic display, significant terms were sorted by FDR, decreasing fold enrichment and term name. A term was retained only when its gene-set Jaccard similarity was below 0.50 to every already retained term, with at most eight terms per query-library combination. A separately frozen concept audit searched significant term names for degranulation, secretion, GM-CSF/JAK-STAT, PI3K/AKT, innate-myeloid, metabolic and stress-survival concepts. Absent concepts were recorded as null rather than replaced by nominal terms.

### Frozen green-module projection into external cohorts

External projection used all 627 green-module members and their original positive GSE76293 Part B PC1 loadings. For each external profile, measured genes were transformed to within-profile percentile ranks. The primary score was

2 × [sum(w_i × r_ij) / sum(w_i) - 0.5],

where r_ij is the percentile rank of mapped gene i in profile j, and w_i is its frozen loading. Loadings were never refitted, rotated or sign-flipped externally. Unweighted all-member and frozen-hub weighted scores were sensitivity analyses. Count-based duplicate symbols were summed, whereas normalized-expression duplicates were collapsed deterministically by their mean.

Formal eligibility required at least 70% coverage of both the 627 genes and total absolute loading mass. GSE151263, GSE240580 and GSE273149 passed this gate. GSE116560 did not and was analysed only as a platform-limited sensitivity. Absolute projected scores were not compared across cohorts.

In GSE151263, eligible pseudobulks required at least 50 cells and 50,000 UMIs. A Friedman test compared the three fully represented myeloid states across seven patients. One-sided paired exact Wilcoxon tests then evaluated the pre-specified low-density-neutrophil-high direction, with Benjamini-Hochberg correction across both comparisons. Disease-group inference was prohibited for this projection.

In GSE240580, baseline broad-neutrophil and monocyte/macrophage profiles were compared within patients using a one-sided exact Wilcoxon signed-rank test. The longitudinal endpoint was the not-resolved-minus-resolved difference in within-patient broad-neutrophil score change. Its one-sided P value used the exact patient-label allocation distribution, and stability was assessed by leaving out each patient.

In GSE273149, Ensembl features were mapped to canonical symbols and duplicate count rows were summed. A separate weighted-score slope was estimated for each of the nine COVID-19 ARDS patients. The non-survivor-minus-survivor slope contrast used the same 126-allocation exact one-sided test and leave-one-patient-out audit. Because outcomes had already been inspected for the anchor analysis, this projection was designated post hoc external exploratory evidence. The two eligible longitudinal P values from GSE240580 and GSE273149 were Benjamini-Hochberg adjusted together.

GSE116560 used the existing random-intercept mixed-model structure but failed the coverage gate. Its results could not rescue an unsupported eligible-cohort endpoint. No external cohort was used to modify green membership, loadings, thresholds or interpretation rules.

### Contextual boundary analyses

GSE89953 official GPL6883 signals were log2-transformed without repeat between-array normalization. A probe was retained when its detection P value was below 0.05 in at least six of 26 arrays in either compartment. Single-symbol probes were collapsed by gene-wise median. Programme scores were compared between paired alveolar macrophages and peripheral-blood monocytes using paired Wilcoxon tests, with correction across four programmes. Byte-level comparison established overlap with GSE116560, so these analyses were considered within-cohort compartmental context rather than replication.

GSE32707 raw GPL10558 signals underwent norm-exponential background correction with offset 16, log2 transformation and quantile normalization. Technical replicates were averaged after normalization. The main track contained 85 patient-level units, and a separately normalized strict-QC sensitivity contained 82. Programme contrasts used fixed group designs and limma; this whole-blood cohort was not used to optimize programme membership or train a classifier.

The GSE207077 submitted matrix was treated as continuous transformed expression rather than raw counts. Duplicate symbols were collapsed by within-sample median, and rank scores were computed without repeat normalization. COVID-19 and influenza ARDS were compared using two-sided Wilcoxon rank-sum tests, with Benjamini-Hochberg correction across four programmes. Welch confidence intervals and Cliff's delta were descriptive. A quality sensitivity excluded one multimetric outlier from each aetiology group.

### Statistical analysis and reproducibility

Tests were two-sided unless a directional alternative was fixed before result inspection. Exact tests were used when permitted by the design and sample size. Paired designs were preserved throughout, repeated samples were not treated as independent observations and no missing expression value was imputed. Paired standardized effects were calculated as the mean within-unit difference divided by its standard deviation. Leave-one-unit-out analyses evaluated directional stability rather than serving as new hypothesis tests.

Benjamini-Hochberg correction was applied within the explicitly defined families described above. A result was not upgraded because it was nominally significant in a sensitivity analysis. No pooled cross-platform effect, classifier, receiver-operating-characteristic curve or outcome-informed cutoff was estimated. Cohort sizes were fixed by public data availability, and no prospective sample-size calculation was performed.

Analyses were performed in R with cohort-specific validation scripts and deterministic random seeds where stochastic algorithms were required. Key packages included Seurat, harmony, scDblFinder, edgeR, limma, nlme, WGCNA, GEOquery, msigdbr and data.table. Frozen specifications, gene-set snapshots, membership tables, source-data tables, software versions and SHA-256 manifests were retained with the corresponding outputs. Exact R and package versions were recorded in the archived software-version tables for each analysis.

## Results

### Study design separates complementary analytical routes and predefines interpretive boundaries

We organized the multi-cohort analysis by biological compartment and evidentiary role rather than treating all datasets as interchangeable replication cohorts (Fig 1a). GSE151263 localized pre-specified programmes in peripheral blood mononuclear cells (PBMCs), whereas GSE240580 assessed the paired pulmonary course in bronchoalveolar lavage fluid (BALF). GSE116560 contributed a longitudinal alveolar-macrophage analysis of 28-day ventilator-free days (VFD). GSE76293 supplied paired disease and ex vivo perturbation analyses in purified neutrophils. Three additional cohorts defined contextual boundaries: GSE89953 was not independent because its alveolar-macrophage arrays overlapped with GSE116560 Day 1. GSE32707 and GSE207077 delimited whole-blood and viral-aetiology interpretations, respectively. The corresponding overlap, compartment and aetiology audits are reported in S1 Fig.

**Fig 1:**
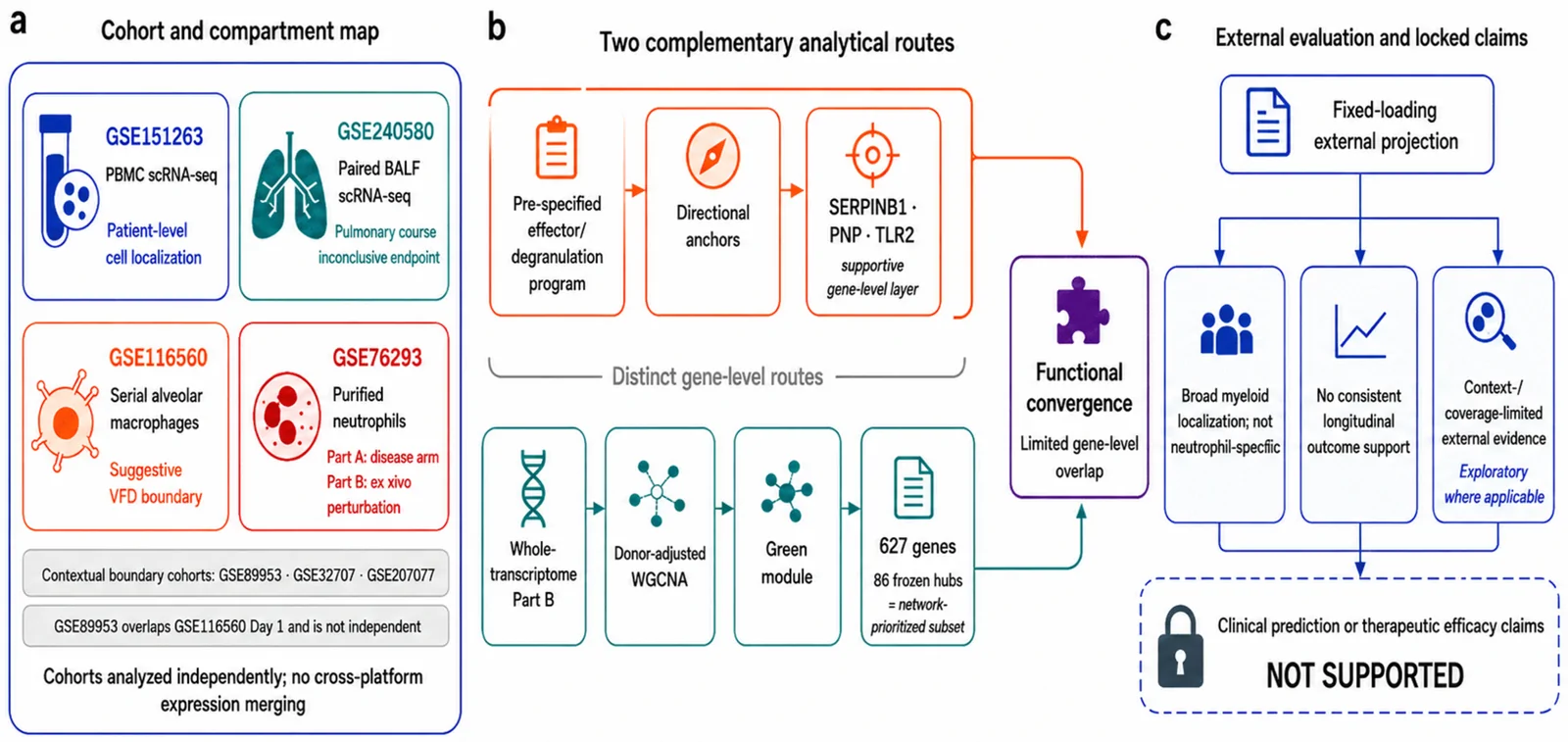
Multi-cohort study design, complementary analytical routes and evidence boundaries. **a, Cohort and compartment map.** GSE151263 was used for patient-level localization of pre-specified programmes in peripheral blood mononuclear cells (PBMCs). GSE240580 assessed the paired pulmonary course in bronchoalveolar lavage fluid (BALF). GSE116560 contributed longitudinal alveolar-macrophage profiles and 28-day ventilator-free days (VFD). GSE76293 supplied paired disease and ex vivo perturbation experiments in purified neutrophils. GSE89953, GSE32707 and GSE207077 defined contextual boundaries. GSE89953 overlapped GSE116560 Day 1 and was not treated as independent evidence. Cohorts were analysed separately, without cross-platform expression merging. **b, Complementary analytical routes.** The hypothesis-driven route tested a pre-specified effector/degranulation programme and retained SERPINB1, PNP and TLR2 as directional response anchors. The data-driven route used whole-transcriptome GSE76293 Part B data for donor-adjusted weighted gene coexpression network analysis (WGCNA). This route retained the 627-gene green module and 86 frozen hubs. The programme was not used as network input, and the anchors were not members of green. The routes shared GSE76293 and were integrated only at the functional level. **c, External evaluation and locked claims.** Fixed-loading projection tested cellular localization, longitudinal outcome support and platform coverage without external refitting. External results supported broad myeloid context but not neutrophil-specific localization or consistent longitudinal outcome association. Context- or coverage-limited findings remained exploratory. The evidence supports association and ex vivo perturbation sensitivity, not a causal programme-to-outcome path, clinical prediction or therapeutic efficacy. This panel is a study schematic and contains no quantitative inferential result.

The analysis followed two complementary gene-level routes (Fig 1b). The hypothesis-driven route tested concordant disease association, GM-CSF induction and broad pan-PI3K reversal within a pre-specified effector/degranulation programme. This route retained SERPINB1, PNP and TLR2 as pre-specified directional response anchors. In parallel, the data-driven route used whole-transcriptome GSE76293 Part B data to construct a donor-adjusted weighted gene coexpression network analysis (WGCNA). The pre-specified programme was not used as network input. This route identified the retained GM-CSF-inducible, pan-PI3K-sensitive coexpression module (green module), which contained 627 genes and 86 frozen hubs. The anchors and green module remained separate throughout. Because both routes used GSE76293, they were not considered independent biological validation and were integrated only at the functional level.

External evaluation was designed to test evidentiary limits rather than refit or optimize each cohort (Fig 1c). Cohorts were processed independently, and absolute scores were not compared across platforms. Green-module projection used frozen loadings and a pre-specified coverage gate. This framework separated disease association and ex vivo perturbation sensitivity from cellular specificity and longitudinal outcome support. Causal programme-to-outcome inference, clinical prediction and therapeutic efficacy remained unsupported without independent outcome replication or orthogonal experiments.

### Patient-level PBMC profiles localize pre-specified programmes to myeloid states

We assessed the cellular distribution of the pre-specified programmes in GSE151263, which contained 23,485 quality-controlled core singlets from seven patients (Fig 2a). Fourteen frozen cell-type and cell-state annotations defined the atlas, whereas programme summaries used patient pseudobulks. The four programmes were highest or among the highest in circulating myeloid states (Fig 2b). These states included CD14 inflammatory monocytes, FCGR3A non-classical monocytes and low-density neutrophils. The mean neutrophil-degranulation score was 0.608 in CD14 inflammatory monocytes and 0.598 in low-density neutrophils. Corresponding values across the principal lymphoid states ranged from 0.363 to 0.395. This seven-patient PBMC cohort provided descriptive myeloid cell-state context for the pre-specified programmes; it did not test an ARDS-specific signature.

**Fig 2:**
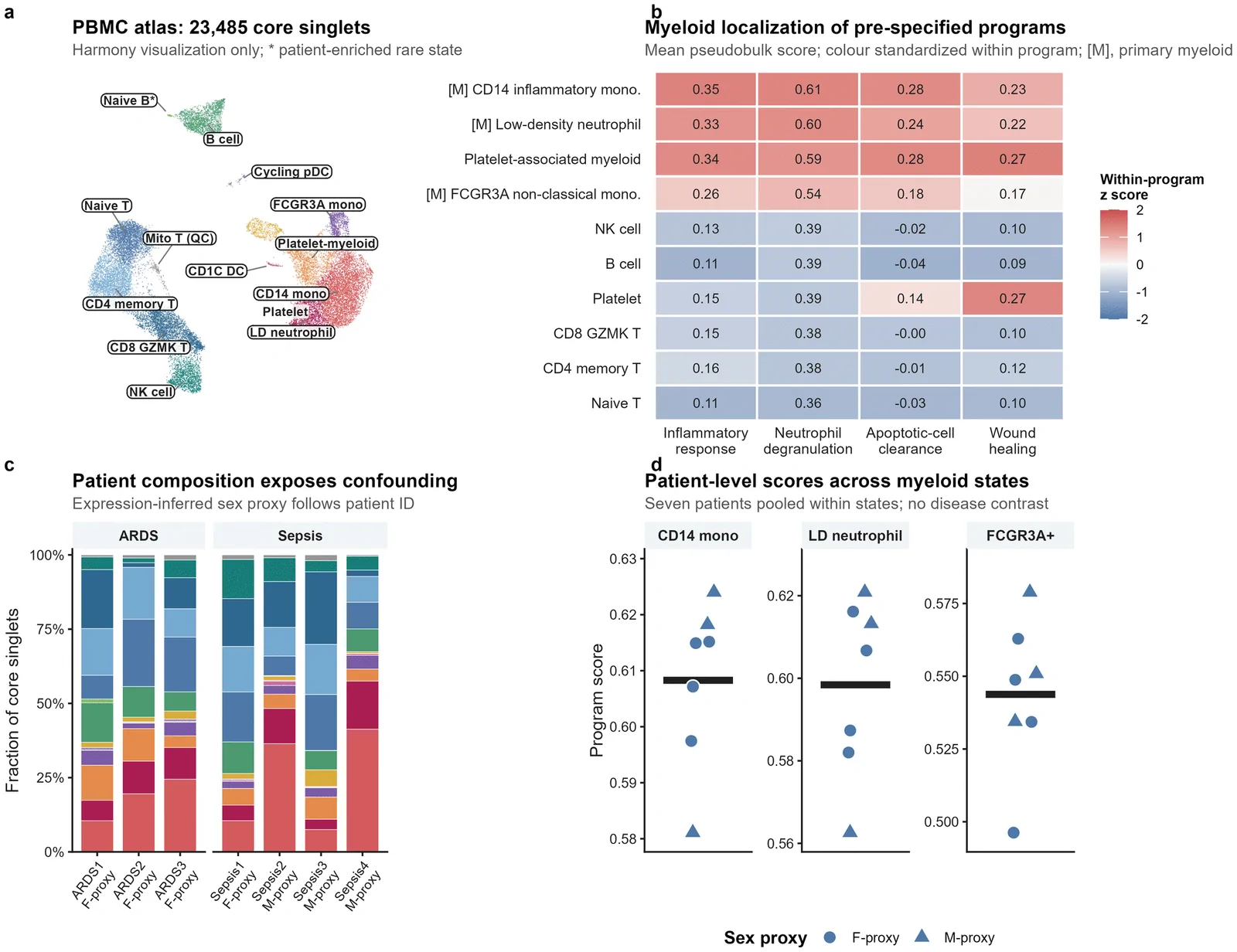
Patient-level PBMC profiles localize pre-specified programmes to myeloid states. **a,** Uniform manifold approximation and projection (UMAP) of 23,485 quality-controlled core singlets from seven patients, coloured by 14 frozen cell-type and cell-state annotations. Harmony coordinates are shown only for visualization and were not used for differential-expression inference. The rare naive-B-cell state was enriched in one patient. **b,** Localization of four pre-specified external programmes across eligible PBMC states. Values are mean patient-pseudobulk programme scores. Colour denotes the within-programme z score across cell states and is not an absolute scale for between-programme comparison. [M] marks the three pre-specified primary myeloid states. **c,** Patient-level cell-state composition for three patients with acute respiratory distress syndrome (ARDS) and four with sepsis. All three ARDS patients and one sepsis patient had a female expression-inferred sex proxy; the other three sepsis patients had a male proxy. Proxies were not independently verified demographic sex. Disease labels show cohort composition only. **d,** Patient-level neutrophil-degranulation programme scores in the three primary myeloid states. Points represent patients, shapes denote expression-inferred sex proxies and horizontal bars show pooled across-patient means within each state. Disease groups are neither separated nor tested in this panel; panel c displays the confounding. Patients are the unit of observation, and the cell-level atlas is descriptive.

GSE151263 did not permit ARDS–sepsis inference. All three ARDS patients had female expression-inferred sex proxies; three of four sepsis patients had male proxies (Fig 2c). Proxy sex was not independently verified. This seven-patient imbalance makes disease attribution impossible. Figure 2d therefore pools individual scores by cell state. Archived ARDS–sepsis tests and their nonsignificant P values are not negative evidence. The cohort contributes cell-state localization only.

### Paired BALF provides pulmonary context and an inconclusive longitudinal test

We next tested the pre-specified neutrophil-degranulation programme in paired GSE240580 BALF samples. The dataset contained 87,115 core singlets from 16 libraries representing eight patients sampled twice. The cell atlas provided pulmonary context, whereas inference used patient-level neutrophil pseudobulks (Fig 3a). Five patients had clinical remission (resolved), whereas three had non-remission (not resolved), at the second sampling point. The proposed non-resolution direction predicted greater persistence or increase among patients with non-remission. Instead, the non-remission-minus-remission contrast in within-patient change was −0.0147 (two-sided exact Wilcoxon rank-sum P = 0.571; Fig 3b). In this eight-patient cohort, the estimate opposed the hypothesis but was inconclusive.

**Fig 3:**
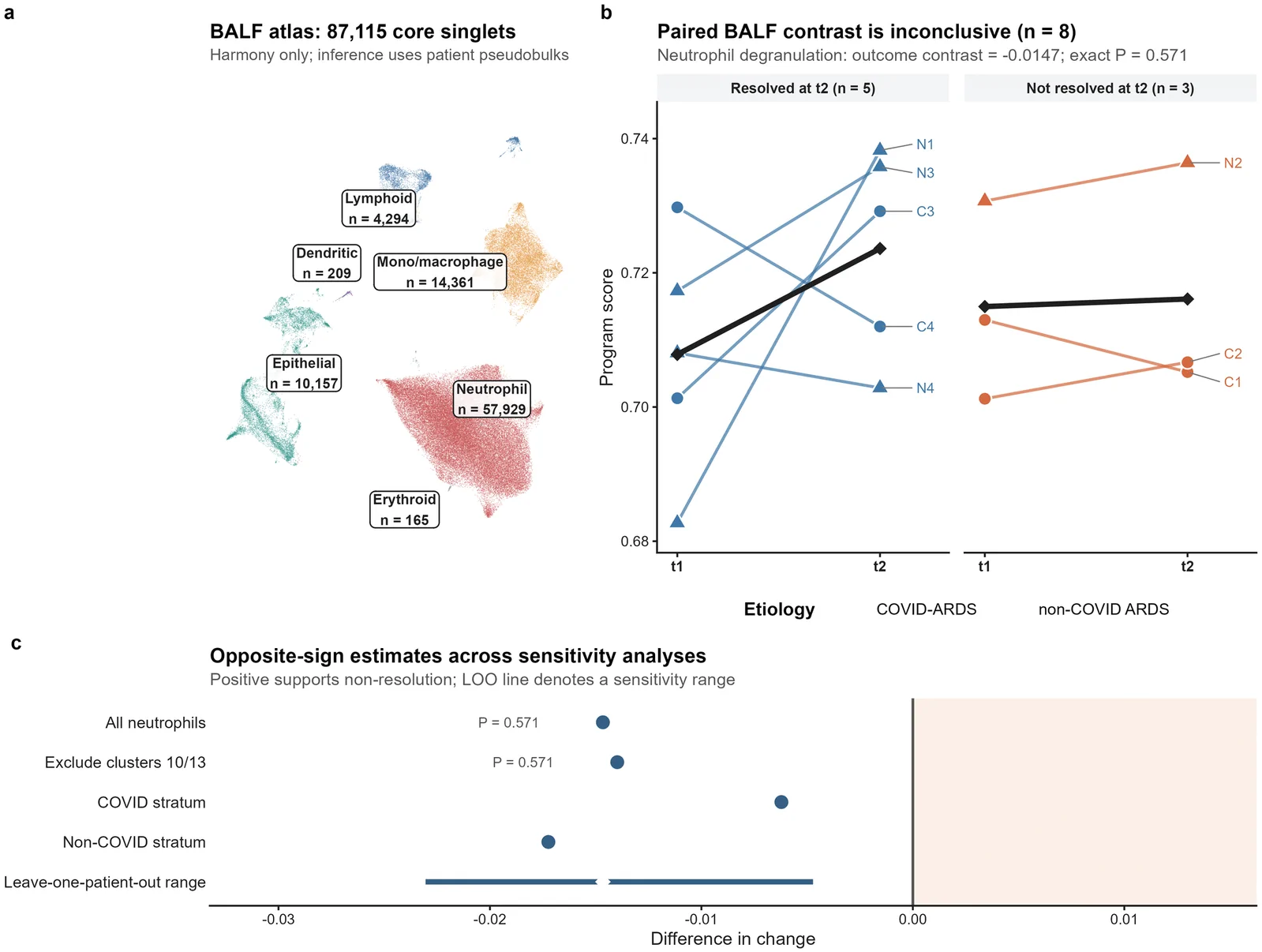
Paired BALF provides pulmonary context and an inconclusive longitudinal test. **a,** Harmony embedding of 87,115 core BALF singlets from 16 libraries representing eight patients sampled at two time points. Cells provide descriptive pulmonary context and are labelled by broad cell class. All inference used patient-level pseudobulks. **b,** Paired patient-level neutrophil-degranulation programme scores at baseline (t1) and follow-up (t2), stratified by clinical remission at t2. Five patients had remission (resolved), whereas three had non-remission (not resolved). Lines connect observations from the same patient, shapes denote COVID-19 or non-COVID-19 ARDS, and black lines and diamonds denote group means. The pre-specified non-remission-minus-remission contrast in within-patient change was −0.0147 (two-sided exact Wilcoxon rank-sum P = 0.571). No multiplicity correction was applied to this single pre-specified primary test. **c,** Planned sensitivity analyses of the primary outcome contrast. Positive values would support greater programme persistence or increase among patients with non-remission. Points show the complete neutrophil analysis, exclusion of patient-enriched clusters 10 and 13, and aetiology-stratified estimates. The horizontal line shows the leave-one-patient-out range and is not a confidence interval. All displayed estimates remained negative. The sampling interval between t1 and t2 was not reported. Patients were the statistical unit.

Estimates retained the opposite sign across planned sensitivity analyses (Fig 3c). Excluding patient-enriched neutrophil clusters 10 and 13 produced a contrast of −0.0140 (exact P = 0.571). COVID-19 and non-COVID-19 strata also remained negative (−0.00622 and −0.0172, respectively). Leave-one-patient-out contrasts ranged from −0.0230 to −0.00472 and remained below zero in every iteration. An exploratory baseline monocyte/macrophage wound-healing contrast yielded −0.0303 (exact P = 0.0357), but its BH-FDR was 0.286. The difference was absent at follow-up (contrast, 0.00487; exact P = 1.000; BH-FDR = 1.000; S2 Fig). This exploratory result did not alter the pre-specified neutrophil-degranulation endpoint. Because the cohort used BALF and categorical resolution, this small, endpoint-specific test cannot determine whether the proposed longitudinal direction generalizes.

### A longitudinal alveolar-macrophage trajectory remains suggestive within a single cohort

We separately assessed longitudinal alveolar macrophage (AM) profiles from GSE116560 in relation to 28-day VFD. The dataset contained 68 samples from 35 patients, analysed with patient-specific random intercepts. Sampling declined from 30 samples on Day 1–22 on Day 4 and 16 on Day 8, with outcome-imbalanced later availability (Fig 4a). At Day 8, only five samples came from patients with VFD > 0, compared with 11 from patients with VFD = 0. Raw trajectories suggested a greater mean programme increase in the VFD = 0 group (Fig 4b). These binary groups were descriptive; the pre-specified model treated VFD as continuous.

**Fig 4:**
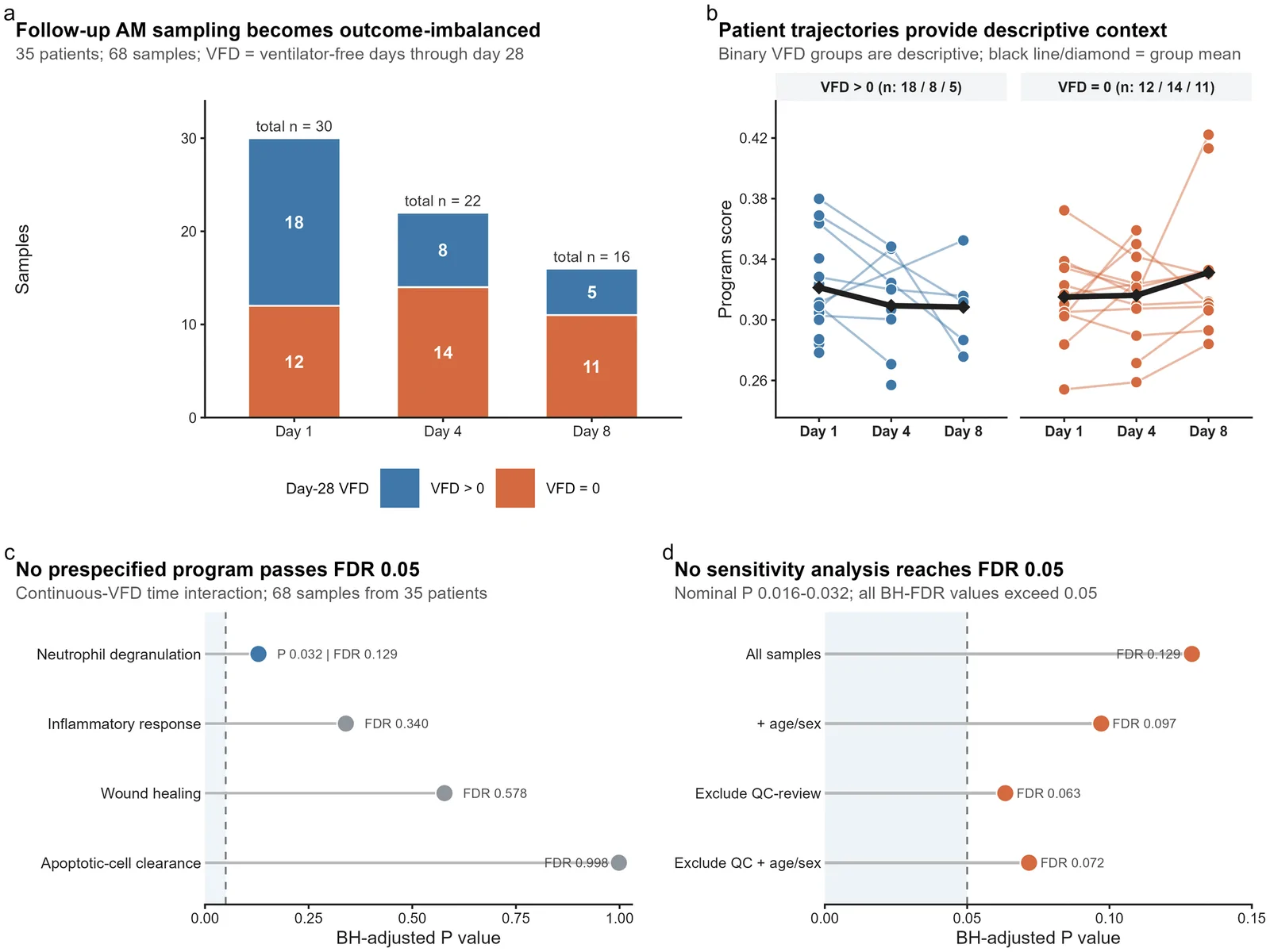
A longitudinal alveolar-macrophage trajectory remains suggestive within a single cohort. **a,** Longitudinal sampling of 68 alveolar-macrophage (AM) profiles from 35 patients on Days 1, 4 and 8. Bar segments represent samples from patients with 28-day VFD greater than zero or equal to zero. Sampling became outcome-imbalanced. At Day 8, five samples were available from patients with VFD > 0 and 11 from patients with VFD = 0. **b,** Raw patient-level neutrophil-degranulation programme scores over time, displayed by descriptive binary VFD group. Lines connect samples from the same patient, and black lines and diamonds denote group means. The pre-specified primary model treated VFD as continuous; the binary groups were used only to display raw trajectories. **c,** BH-adjusted P values for the time-by-continuous-VFD interaction across four pre-specified programmes. The linear mixed model included patient-specific random intercepts and all 68 samples from 35 patients. No programme met BH-FDR < 0.05. The neutrophil-degranulation interaction had nominal P = 0.0322 and BH-FDR = 0.1289. **d,** Sensitivity analyses of the continuous-VFD neutrophil-degranulation interaction. Tracks included age and sex adjustment, exclusion of six quality-control-review samples, and both changes together. Nominal P values ranged from 0.0158 to 0.0322, whereas BH-FDR values ranged from 0.0634 to 0.1289. No sensitivity analysis met BH-FDR < 0.05. The cohort provides directionally suggestive single-cohort evidence, not a validated prognostic association.

The primary time-by-continuous-VFD interaction yielded P = 0.0322 and was directionally consistent with greater persistence among patients with fewer VFD. It did not pass correction across the four pre-specified programmes (BH-FDR = 0.1289; Fig 4c). The remaining BH-FDRs were 0.340 for inflammatory response, 0.578 for wound healing and 0.998 for apoptotic-cell clearance. Neutrophil-degranulation sensitivity analyses produced nominal P values of 0.0158–0.0322 after age/sex adjustment, QC exclusion, or both. Their BH-FDRs ranged from 0.0634 to 0.1289 and remained above 0.05 (Fig 4d). GSE116560 therefore provided a directionally suggestive single-cohort AM/VFD trajectory, but outcome-dependent follow-up, multiplicity and absent independent replication precluded prognostic interpretation.

### ARDS neutrophils show a GM-CSF-inducible, pan-PI3K-sensitive effector programme

GSE76293 provided the strongest disease-association and perturbation evidence through two distinct paired experiments (Fig 5a). Part A compared neutrophils from 12 patients with ARDS with matched healthy-volunteer neutrophils. Part B used a separate set of ten healthy donors to assess 6-h GM-CSF exposure and addition of a broad pan-PI3K inhibitor. Comparisons were paired within each experiment, and Part A and Part B were not treated as one biological sample set.

**Fig 5:**
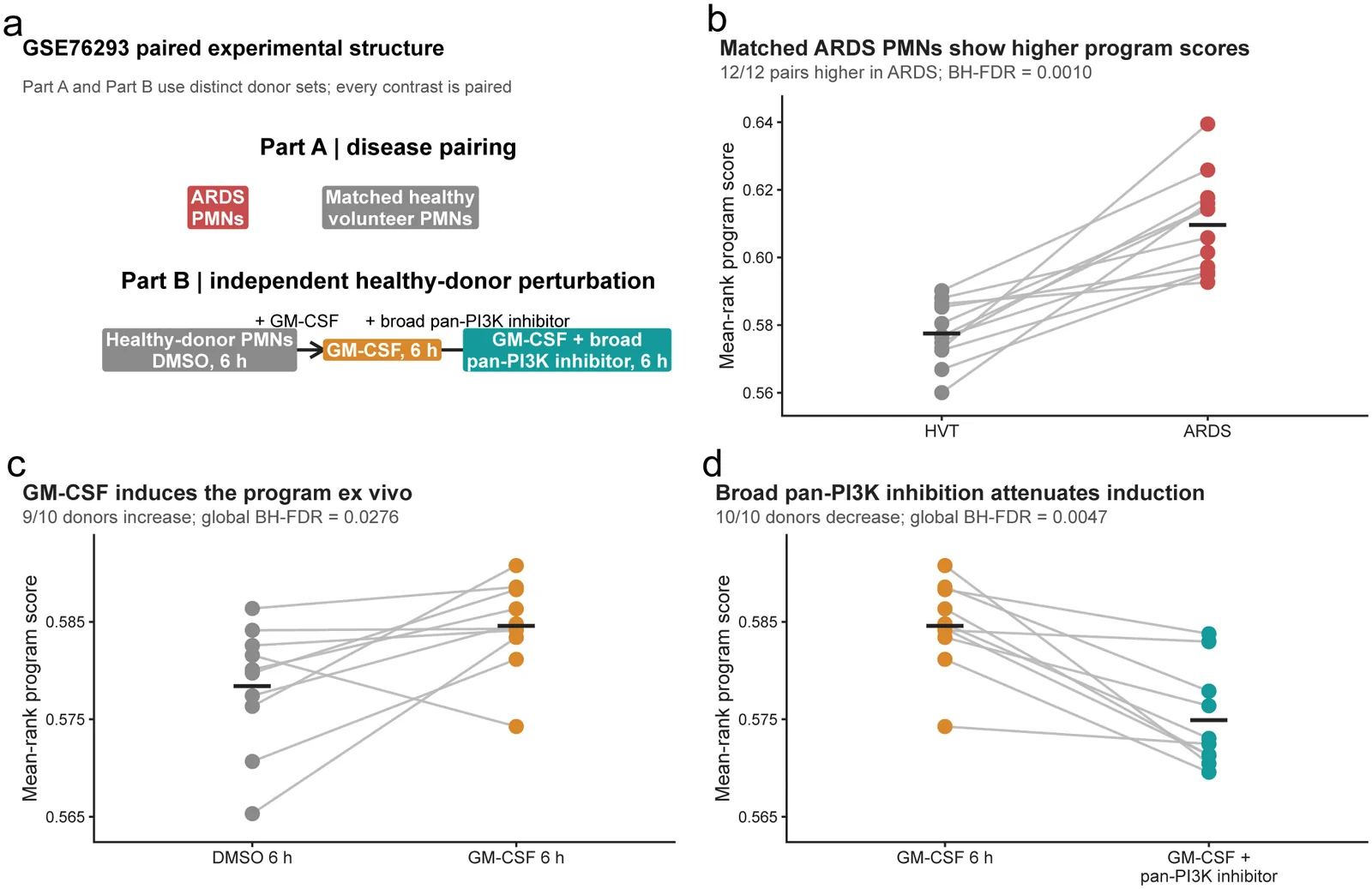
ARDS neutrophils show a GM-CSF-inducible, broad pan-PI3K-sensitive effector programme. **a,** Paired experimental structure of GSE76293. Part A compared neutrophils from 12 patients with ARDS with matched healthy-volunteer neutrophils. Part B was a separate experiment in ten healthy donors. Conditions comprised 6-h exposure to dimethyl sulfoxide (DMSO), granulocyte-macrophage colony-stimulating factor (GM-CSF), or GM-CSF plus a broad pan-phosphoinositide 3-kinase (pan-PI3K) inhibitor. Part A and Part B used distinct donor sets, and comparisons were paired within each experiment. **b,** Pre-specified neutrophil-degranulation programme scores in 12 matched ARDS-healthy-volunteer pairs. Lines connect matched samples, and horizontal black marks denote group means. All 12 pairs had higher scores in ARDS neutrophils. The mean paired difference was 0.0321 (two-sided Wilcoxon signed-rank P = 4.88 × 10⁻⁴; BH-FDR = 9.77 × 10⁻⁴). **c,** Programme scores after DMSO or GM-CSF exposure in ten Part B donors. Nine of ten donors had higher scores after GM-CSF. The mean paired difference was 0.00618 (two-sided Wilcoxon signed-rank P = 0.0195; global BH-FDR = 0.0276). **d,** Programme scores after GM-CSF alone or GM-CSF plus broad pan-PI3K inhibition in the same ten donors. All ten donors had lower scores after inhibitor addition. The mean inhibitor-minus-GM-CSF difference was −0.00968 (two-sided Wilcoxon signed-rank P = 0.00195; global BH-FDR = 0.00469). The inhibitor result supports broad ex vivo pan-PI3K sensitivity. The final DMSO percentage and condition-matched 6-h viability were not verifiable; the contrast does not establish PI3K isoform specificity, a viability-independent mechanism or therapeutic efficacy. The matched pair was the biological unit in Part A, and the donor was the biological unit in Part B.

The pre-specified neutrophil-degranulation programme was higher in ARDS neutrophils in all 12 matched pairs (Fig 5b). The mean paired difference was 0.0321 (two-sided Wilcoxon signed-rank P = 4.88 × 10⁻⁴; BH-FDR = 9.77 × 10⁻⁴). In Part B, GM-CSF increased the programme score in nine of ten donors relative to DMSO (Fig 5c). The mean paired difference was 0.00618 (Wilcoxon signed-rank P = 0.0195; global BH-FDR = 0.0276). Adding the broad pan-PI3K inhibitor reduced the score in all ten donors (Fig 5d). The mean inhibitor-minus-GM-CSF difference was −0.00968 (P = 0.00195; global BH-FDR = 0.00469). These results supported disease association, ex vivo GM-CSF inducibility and broad pan-PI3K sensitivity at the programme level. They did not establish PI3K isoform specificity, therapeutic efficacy or a complete causal mechanism.

We then tested whether the three concordant directions defined a genome-wide gene-level core. Across the genome, 5,585 genes had positive effects in all three directions, and 251 had three one-sided P values below 0.05. No gene passed the intersection–union conjunction threshold at BH-FDR ≤ 0.05; the minimum BH-FDR was 0.116 (S3 Fig). Restricting multiplicity to detected members of the pre-specified programme retained SERPINB1, PNP and TLR2 under the frozen rule. These genes were designated directional response anchors, not a genome-wide core or green-module members. Their paired GSE76293 effects were concordant, but their frozen GSE273149 score produced a non-survivor-minus-survivor slope difference of 0.00224 per day. The one-sided exact patient-label permutation P was 0.2143, and the leave-one-patient-out positive-direction fraction was 1.000 (S4 Fig). This result was direction-only/inconclusive and did not constitute outcome validation or pulmonary replication.

### Donor-adjusted WGCNA identifies one retained perturbation-response module

In a deterministic secondary analysis, we constructed a donor-adjusted WGCNA from the 60 GSE76293 Part B arrays. The design comprised ten healthy donors across six paired conditions. The pre-specified degranulation programme was not used as network input. An outcome-blind, top-half donor-adjusted median-absolute-deviation filter retained 10,827 of 21,653 input genes. Donor and RNA integrity number effects were adjusted while preserving condition means. Power 8 was the first signed-network candidate with scale-free-fit R^2^ ≥ 0.80 and yielded 13 non-grey modules (Fig 6a).

**Fig 6:**
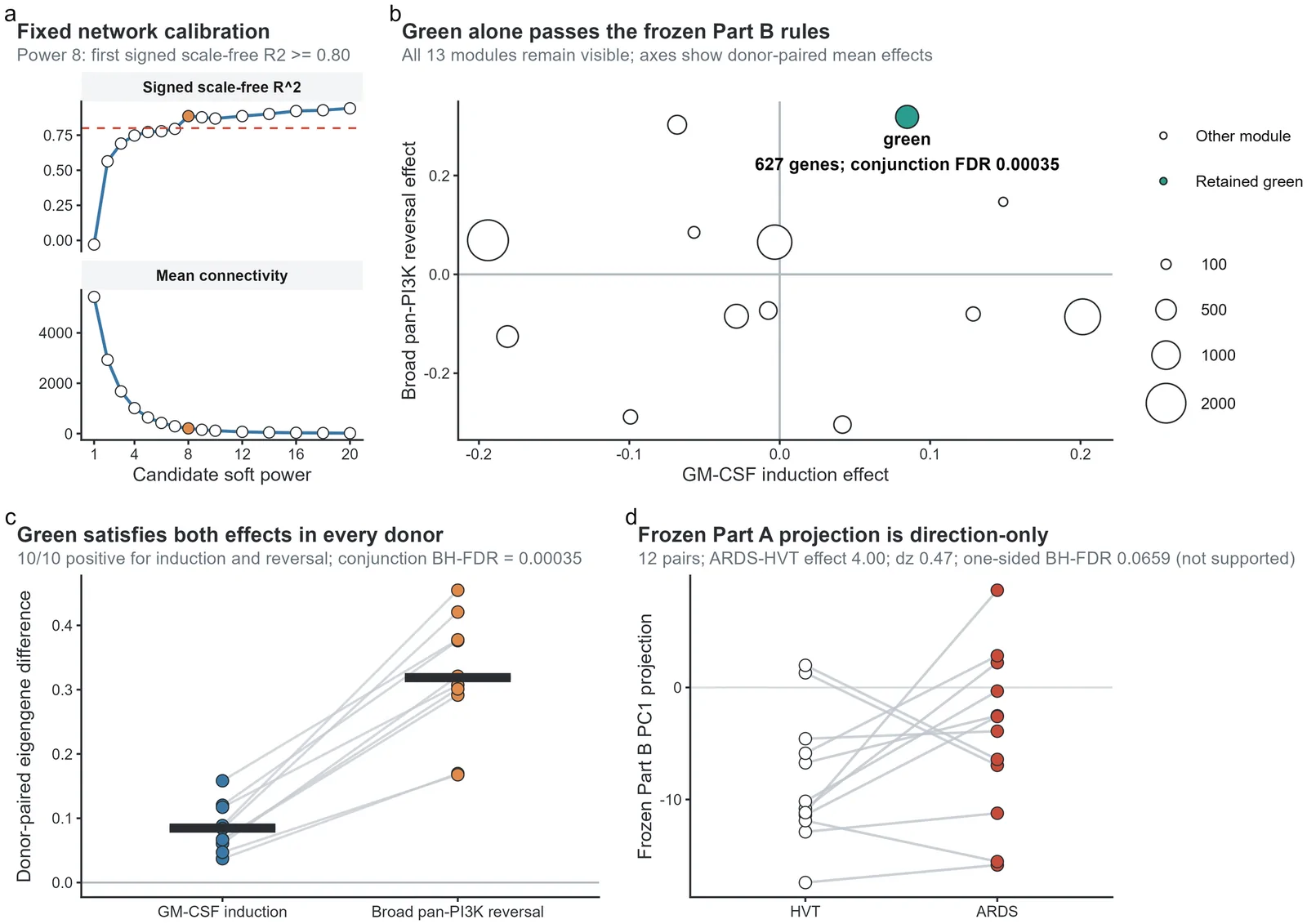
Donor-adjusted WGCNA identifies one retained perturbation-response module. **a,** Deterministic calibration of the signed donor-adjusted Part B network. The input comprised 60 arrays from ten donors across six paired conditions. Power 8 was the first candidate with signed scale-free-fit R^2^ ≥ 0.80 and was fixed before module-level perturbation testing. **b,** Thirteen non-grey modules positioned by their paired mean GM-CSF-induction and broad pan-PI3K-reversal effects. Point area denotes module size. Green, containing 627 genes, was the only module meeting every frozen Part B criterion (intersection-union conjunction BH-FDR = 3.50 × 10⁻⁴). **c,** Donor-level green-module eigengene differences for GM-CSF induction and broad pan-PI3K reversal. Lines connect the two contrast estimates from each donor for compact display, and horizontal black marks denote means. Both effects were positive in all ten donors. The mean induction effect was 0.0847 (paired d^z^ = 2.26), and the mean reversal effect was 0.3187 (paired d^z^ = 3.34). **d,** Frozen Part B first principal-component (PC1) projection into the 12 matched Part A ARDS-healthy-volunteer pairs without loading refitting. Nine of 12 pairs had higher projected scores in ARDS. The mean projected-score difference was 4.00 (paired d^z^ = 0.47; one-sided BH-FDR = 0.0659), which did not meet the frozen Part A support threshold. Green is a Part B perturbation-response module with 627 members and 86 frozen hubs. Part A provides direction-only within-accession evidence and is not external validation. SERPINB1, PNP and TLR2 belong to a separate analytical route and are not green-module members.

Green alone met all frozen criteria for GM-CSF induction and broad pan-PI3K reversal (Fig 6b). The 627-gene module had a mean induction effect of 0.0847 (paired d^z^ = 2.26). Its mean reversal effect was 0.3187 (paired d^z^ = 3.34). Both effects were positive in all ten donors, and the conjunction BH-FDR was 3.50 × 10⁻⁴ (Fig 6c). Sequential application of the frozen rules reduced the 13 modules to green alone. Adjacent-power membership was stable, with Jaccard indices of 0.839 at power 7 and 0.838 at power 9 (S5 Fig). The balanced design and deterministic diagnostics supported technical robustness, not independent biological validation.

Green contained 627 members and 86 frozen hubs. Hubs required oriented module membership (kME) ≥ 0.70, positive mean effects and positive donor fractions of at least 0.70 in both contrasts. These Part B-only rules did not use Part A or external outcomes (S6 Fig). We projected the frozen first principal-component (PC1) loadings into the 12 matched Part A ARDS–healthy-volunteer pairs without refitting. Nine pairs had higher projected scores in ARDS, with a mean projected-score difference of 4.00 (paired d^z^ = 0.47). The one-sided BH-FDR was 0.0659, above the frozen 0.05 threshold (Fig 6d). Green was therefore retained as a Part B perturbation-response module with direction-only within-accession Part A evidence. It was not an FDR-supported ARDS module or externally validated outcome module.

### Separate analytical routes converge functionally despite limited gene-level overlap

We compared the pre-specified programme/anchor route with the data-driven green-module route while preserving their distinct assignments. SERPINB1 and TLR2 mapped to the blue WGCNA module, which did not meet the frozen perturbation-response criteria, whereas PNP was grey and unassigned. None of the directional response anchors belonged to green (Fig 7a). The anchors and green module therefore represented separate gene-level outputs rather than one core recovered by different methods.

**Fig 7:**
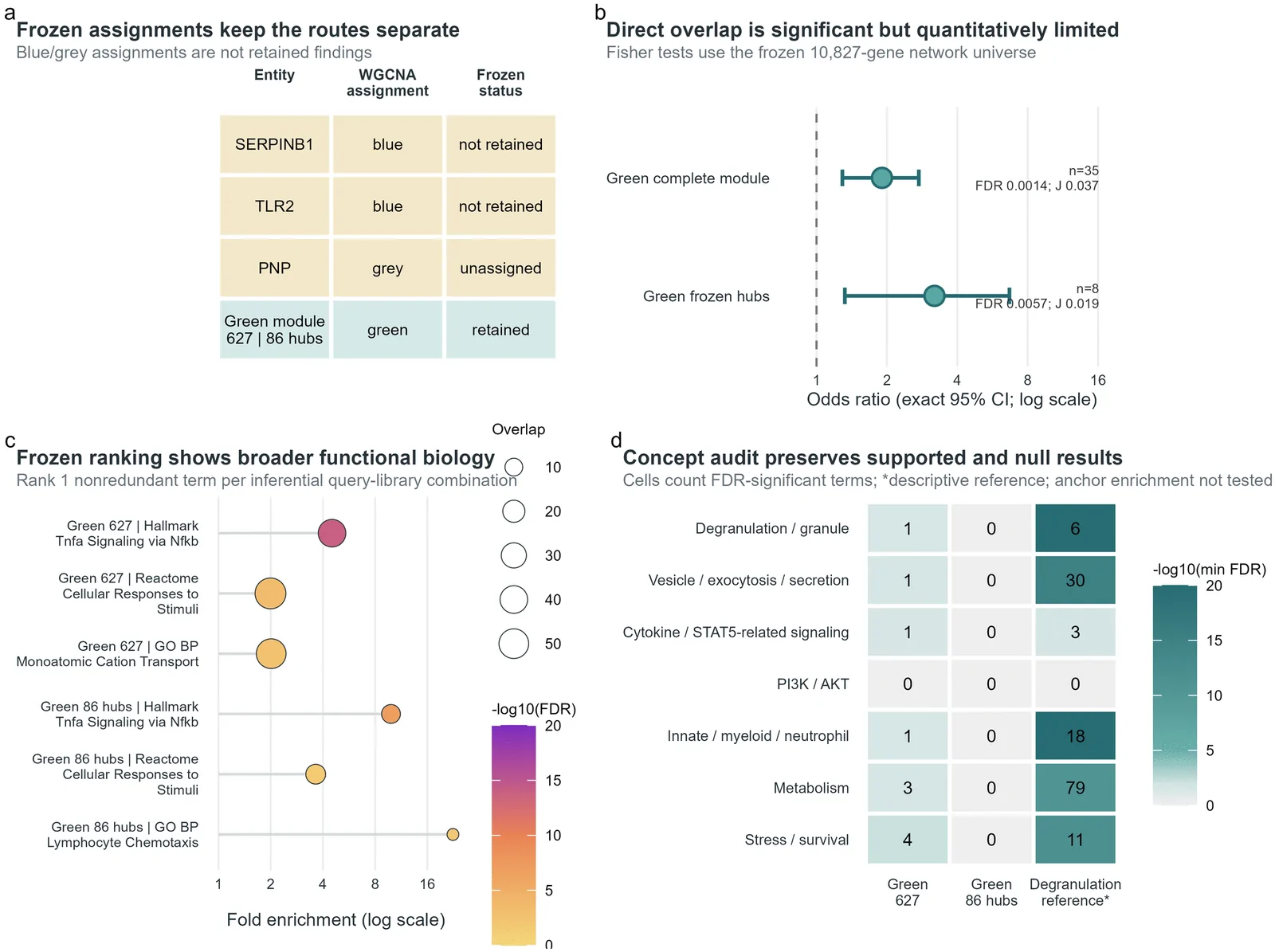
Separate analytical routes converge functionally despite limited gene-level overlap. **a,** Frozen gene assignments preserve separation of the two analytical routes. SERPINB1 and TLR2 mapped to the blue WGCNA module, which was not retained, whereas PNP was grey and unassigned. None of the directional response anchors belonged to the retained green module. **b,** One-sided Fisher's exact enrichment tests compared the complete green module and its frozen hubs with the pre-specified neutrophil-degranulation reference set over the fixed 10,827-gene network universe. Green shared 35 of 627 genes with 342 mapped reference genes (odds ratio, 1.905; exact 95% confidence interval, 1.290–2.739; BH-FDR = 0.00144; Jaccard index, 0.0375). The 86 frozen hubs shared eight reference genes (odds ratio, 3.195; exact 95% confidence interval, 1.322–6.685; BH-FDR = 0.00569; Jaccard index, 0.0190). **c,** Frozen-rule over-representation analysis. One rank-1 redundancy-pruned term is shown for each inferential query-by-library combination. Point position denotes fold enrichment, point area denotes overlap count and colour denotes BH-FDR. The display rule was fixed before figure composition and was not manually restricted to favourable terms. **d,** Pre-specified functional concept audit. Cells show the number of significant mapped terms for the complete green module, frozen green hubs and descriptive degranulation reference. The reference column is descriptive, and anchor-set enrichment was not tested. All seven pre-specified concepts are shown, including the null canonical PI3K/AKT result. Significant overlap coexists with low Jaccard indices. The analyses support functional convergence with limited gene-level overlap, not identical membership, shared discovery, causal validation or outcome prediction.

Nevertheless, green showed statistically supported but quantitatively limited overlap with the pre-specified neutrophil-degranulation reference set (Fig 7b). Fisher's exact tests used the fixed 10,827-gene network universe. Thirty-five of 627 green genes overlapped the 342 mapped reference genes (odds ratio, 1.905; exact 95% CI, 1.290–2.739; BH-FDR = 0.00144). The Jaccard index was only 0.0375. The 86 frozen hubs contained eight reference genes (odds ratio, 3.195; exact 95% CI, 1.322–6.685; BH-FDR = 0.00569). Their Jaccard index was 0.0190. These enrichments did not imply gene-level equivalence or establish that the directional anchors were green-module hubs.

Frozen over-representation analysis localized the convergence to functional similarity (Fig 7c, 7d). Green was enriched for Reactome neutrophil degranulation (35 genes; fold enrichment, 1.767; BH-FDR = 0.0293). It was also enriched for Gene Ontology positive regulation of secretion (21 genes; fold enrichment, 2.325; BH-FDR = 0.0254). The broader profile included inflammatory, stimulus-response, stress/homeostatic, metabolic and chemotactic biology. Hallmark IL2–STAT5 signalling passed correction (BH-FDR = 0.0324) but supported only cytokine/STAT5-related signalling; no canonical PI3K/AKT term passed the frozen concept audit. S7 Fig shows the complete deterministic top-three nonredundant profile. The hub subset remained a network-prioritization layer rather than a separate validation result. These analyses supported functional convergence with limited gene-level overlap, not shared discovery, causal validation or outcome prediction.

### Fixed-loading projection limits cellular specificity and provides no consistent outcome support

We projected all 627 green genes into external cohorts using frozen GSE76293 Part B PC1 loadings and within-profile percentile ranks. No external refitting, loading rotation or gene reselection was performed. Eligibility required at least 70% coverage for both mapped genes and loading mass (Fig 8a); GSE151263, GSE240580 and GSE273149 met this gate. Their gene coverage was 87.4%, 87.7% and 86.6%, and loading-mass coverage was 89.5%, 89.5% and 88.5%, respectively. GSE116560 covered only 60.6% of genes and 63.6% of loading mass and remained platform-limited exploratory evidence. Only within-cohort contrasts were interpreted; absolute scores were not compared across platforms.

**Fig 8:**
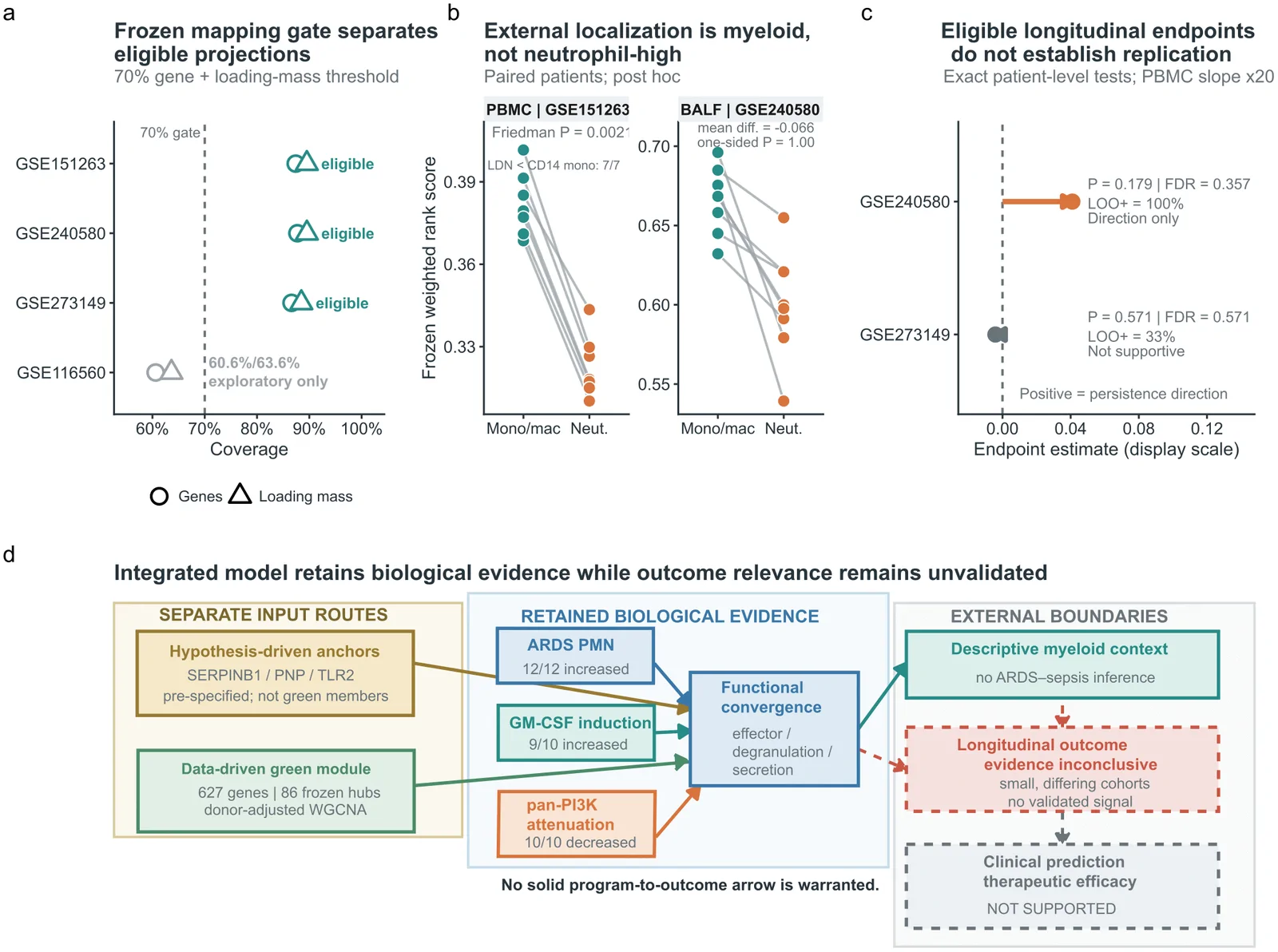
Fixed-loading projection bounds cellular specificity and outcome interpretation. **a,** Frozen external-projection coverage gate. All 627 green-module members were projected using fixed GSE76293 Part B PC1 loadings and within-profile percentile ranks. Eligibility required at least 70% coverage of both mapped genes and loading mass. GSE151263, GSE240580 and GSE273149 met both thresholds. GSE116560 covered 60.6% of genes and 63.6% of loading mass and remained platform-limited exploratory evidence. **b,** External cellular localization. Frozen weighted scores are paired within patients for monocyte/macrophage and neutrophil states in PBMC and BALF. The three-state GSE151263 myeloid comparison was heterogeneous (Friedman P = 0.00215), and low-density-neutrophil scores were below CD14 inflammatory-monocyte scores in all seven patients. In baseline GSE240580 BALF, neutrophil scores were below monocyte/macrophage scores in all eight patients. The mean paired difference was −0.0655 (one-sided exact Wilcoxon signed-rank P = 1.000 for the pre-specified neutrophil-high direction). These localization analyses are post hoc and hypothesis-generating. **c,** Eligible longitudinal endpoints. The GSE240580 non-remission-minus-remission change estimate was 0.0410 (one-sided exact patient-label permutation P = 0.1786; BH-FDR = 0.3571; leave-one-out positive direction, 100%) and was classified as direction-only/inconclusive. The GSE273149 non-survivor-minus-survivor patient-slope estimate was −0.000208 per day (one-sided exact P = 0.5714; BH-FDR = 0.5714; leave-one-out positive direction, 33%) and was classified as not supportive. The GSE273149 estimate was multiplied by 20 for display only. This scaling does not change the estimate or permit cross-cohort effect comparison. Neither endpoint establishes longitudinal outcome replication. **d,** Integrated evidence model. The 12/12 ARDS increase, 9/10 GM-CSF induction and 10/10 pan-PI3K reversal counts describe programme-level evidence and are not three-gene results. SERPINB1, PNP and TLR2 form a separate directional-anchor layer and are not retained green-module members. The data-driven green-module route remains analytically separate, and the routes converge only at the functional level. External projection provides descriptive myeloid cell-state context, not GSE151263 ARDS–sepsis inference, given severe disease–sex-proxy confounding. The post hoc monocyte/macrophage ordering does not establish neutrophil specificity. Eligible longitudinal tests were inconclusive or not supportive and did not validate an outcome signal. Clinical prediction, a causal outcome mechanism and therapeutic efficacy are not supported. Abbreviations: AM, alveolar macrophage; ARDS, acute respiratory distress syndrome; BALF, bronchoalveolar lavage fluid; BH-FDR, Benjamini-Hochberg false-discovery rate; DMSO, dimethyl sulfoxide; GM-CSF, granulocyte-macrophage colony-stimulating factor; PBMC, peripheral blood mononuclear cell; PC1, first principal component; PI3K, phosphoinositide 3-kinase; UMAP, uniform manifold approximation and projection; VFD, ventilator-free days; WGCNA, weighted gene coexpression network analysis.

Eligible cellular projections did not support a neutrophil-high pattern (Fig 8b). In GSE151263, the three-state patient-level myeloid comparison was heterogeneous (Friedman P = 0.00215). Low-density-neutrophil scores were below CD14 inflammatory-monocyte scores in all seven patients. In baseline GSE240580 BALF profiles, neutrophil scores were below monocyte/macrophage scores in all eight patients. The mean paired difference was −0.0655 (one-sided exact Wilcoxon signed-rank P = 1.000 for the pre-specified neutrophil-high direction). Unweighted and hub-only sensitivities retained the same direction. The projection therefore indicated broad myeloid, monocyte/macrophage-biased localization rather than neutrophil specificity. This alternative ordering was identified after projection results were examined and remained post hoc and hypothesis-generating.

Eligible longitudinal endpoints were not consistently supportive (Fig 8c). The GSE240580 non-remission-minus-remission change contrast was 0.0410 (one-sided exact permutation P = 0.1786; BH-FDR = 0.3571). Its leave-one-patient-out positive-direction fraction was 1.000, yielding a direction-only/inconclusive classification. The GSE273149 non-survivor-minus-survivor patient-slope contrast was −0.000208 per day (one-sided exact P = 0.5714; BH-FDR = 0.5714). Its positive-direction fraction was 0.333, and the result was classified as not supportive. This green-module result remained distinct from the separate three-anchor score. The GSE273149 estimate was multiplied by 20 for display in Fig 8c; this scaling did not alter the estimate or permit cross-cohort effect comparison. Nominally compatible GSE116560 sensitivities could not override the frozen coverage gate. Full cellular, longitudinal and coverage projections are provided in S8 Fig.

The integrated model retained four evidence layers (Fig 8d): paired ARDS association, ex vivo GM-CSF/pan-PI3K sensitivity, pathway-restricted directional anchors and the Part B green module. The two analytical routes converged only at the functional level. External projection provided descriptive myeloid cell-state context, not a GSE151263 disease contrast; severe disease–sex-proxy confounding precluded the latter. Post hoc monocyte/macrophage ordering did not establish neutrophil-specific localization, and eligible longitudinal tests were inconclusive or not supportive. The data did not support clinical prediction, a causal outcome mechanism or therapeutic efficacy.

## Discussion

This study defines a bounded transcriptional model of myeloid activation in ARDS. The strongest evidence supports an ARDS-associated neutrophil effector programme that is inducible by GM-CSF and sensitive to broad pan-PI3K inhibition ex vivo. A separate whole-transcriptome analysis identified a coordinated perturbation-response module with related functional features. However, neither analytical route produced consistent longitudinal outcome support across eligible external cohorts. The findings therefore distinguish paired disease association and perturbation biology from unvalidated claims about prognosis, causality or treatment.

The paired GSE76293 experiments provide the clearest biological link in this framework. The pre-specified neutrophil-degranulation programme increased in every matched ARDS pair, linking the signal to purified neutrophils rather than mixed-cell abundance alone. GM-CSF induced the same programme in healthy-donor neutrophils, whereas broad pan-PI3K inhibition reversed it consistently. This pattern identifies a cytokine-responsive effector programme that is sensitive to ZSTK474 ex vivo; it does not isolate a PI3K-dependent mechanism from other drug or viability effects. In the source study's separate survival assay, ZSTK474 reversed GM-CSF-associated survival in healthy-donor cells but did not restore normal apoptosis in ARDS blood neutrophils [17], further limiting transfer of the healthy-donor response to patients. In human neutrophils, GM-CSF has previously activated Akt, and PI3K inhibitors reduced superoxide release, adhesion and cytoskeletal responses [28]. A clinical ARDS study also associated disease onset with an increased circulating neutrophil-elastase-to-elafin ratio [4]. Neither finding establishes that GM-CSF is the dominant in vivo driver of the ARDS state. Reports of altered airway neutrophil activation and NET release in ARDS support biological relevance, but do not validate the present programme as a clinical target [29,30].

The gene-level results argue against reducing this biology to a small universal core. No gene passed the genome-wide three-direction conjunction threshold after correction. SERPINB1, PNP and TLR2 were retained only within the pre-specified degranulation membership and should remain directional response anchors. The data-driven green module captured a different layer of the response. It aggregated coordinated, individually modest effects across 627 genes and was selected without using the pre-specified programme. Its Part A projection was directionally concordant but did not meet the frozen support threshold. Together, these findings favour a distributed response architecture over a compact invariant signature.

Functional convergence between the two analytical routes should also be interpreted quantitatively. The green module was enriched for degranulation and secretion biology, yet its direct overlap with the reference programme remained small. None of the three directional anchors belonged to green. This combination suggests convergence at the level of biological process rather than recovery of an identical gene set. The wider green profile included inflammatory, stress, metabolic and chemotactic features, consistent with a broad perturbation response. Hallmark IL2-STAT5 enrichment supports cytokine-related signalling but is not specific evidence for GM-CSF. Likewise, the absence of canonical PI3K/AKT enrichment prevents pathway annotation from substituting for the experimental inhibitor contrast.

The external projections further show that gene-set identity does not guarantee stable cellular identity across compartments. The pre-specified programmes localized broadly to circulating myeloid states in GSE151263; sex-proxy confounding precluded disease-specific inference. The frozen green-module projection showed post hoc monocyte/macrophage-biased ordering in both PBMC and BALF comparisons. A high neutrophil-degranulation programme score in monocytes summarizes shared transcript ranks; it does not demonstrate neutrophil granule release in those cells. Similarly, a monocyte/macrophage-high green projection shows where the frozen Part B loading pattern is expressed, not that the healthy-donor neutrophil response has the same function in those cells. Existing primary studies show that peripheral and alveolar transcriptional programmes are distinct in ARDS [7]. Longitudinal CITE-seq in acute hypoxemic respiratory failure also resolved dynamically changing alveolar monocyte and macrophage subsets [8]. These studies support a compartment- and composition-sensitive interpretation of rank-based scores. The observations are not contradictory because the programme and green module contain different genes and represent different analytical objects. They also caution against treating pathway names as cell-invariant functional labels. The wound-healing set illustrates this problem, because its meaning differed between neutrophil and monocyte/macrophage contexts. Compartment, cell composition and activation state may each influence a rank-based transcriptional score.

The longitudinal findings place a stronger limit on clinical interpretation. GSE116560 suggested that the alveolar-macrophage degranulation trajectory differed with VFD, but the interaction did not survive correction across programmes. Later sampling was also outcome-imbalanced. The eight-patient paired GSE240580 analysis yielded a directionally opposite but inconclusive BALF-neutrophil estimate, including in planned sensitivity analyses. The three-anchor GSE273149 result was direction-only, whereas the green-module slope contrast was not supportive. The original GSE116560 report similarly described time-dependent AM programmes associated with outcome [16]. The GSE240580 source study described a progression-associated IL1B-high neutrophil state in a small paired BALF cohort [15]. Those source reports frame the biological heterogeneity of the present datasets, but neither is independent validation of our frozen scores. Differences in cell type, outcome definition, sampling interval and disease aetiology may contribute to heterogeneity. Current evidence therefore supports context-dependent longitudinal hypotheses, not a general prognostic signature.

Several design choices strengthen this interpretation. Analyses preserved patients, matched pairs or donors as the biological units. Single-cell data were summarized through patient pseudobulks, avoiding cell-level pseudoreplication. Programme membership, directional tests, WGCNA selection rules and projection coverage gates were frozen before their corresponding evaluations. Negative results, post hoc findings and platform-limited analyses remained visible rather than being removed from the evidence chain. The explicit overlap audit also prevented GSE89953 from being counted as an independent replication of GSE116560. These safeguards reduce outcome-driven optimization and distinguish FDR-supported, directional and exploratory findings.

The study also has important limitations. All cohorts were public datasets assembled for different primary questions, and several were small. GSE151263 contained seven patients with severe disease–sex-proxy confounding and cannot support an ARDS–sepsis contrast; inferred sex was not independently verified. GSE240580 contained eight paired patients, and its sampling interval was unavailable. GSE116560 had declining, outcome-imbalanced follow-up and incomplete green-module coverage. GSE273149 was a small PBMC cohort with substantial clinical heterogeneity. Available treatment information differed across studies and could not be adjusted consistently: seven of eight GSE240580 patients had a reported maximum steroid dose, six of 35 GSE116560 patients had systemic steroid use recorded during their clinical course, and eight of nine GSE273149 COVID-19 ARDS patients received dexamethasone versus none of four non-COVID ARDS controls. GSE116560 also arose from a fish-oil trial, without treatment assignment in the GEO sample annotations used here. Exposure timing relative to every transcriptomic sample and comparable dose data were not available. Cross-platform rank scoring improved portability but did not remove differences in tissue, composition, treatment or assay sensitivity. Absolute scores therefore remained incomparable across cohorts.

GSE76293 Part B used healthy-donor neutrophils ex vivo, not cells sampled during ARDS treatment. The available records did not document the final DMSO percentage, an array-specific rationale for the 10 μM dose or condition-matched 6-h viability. Separate 20-h survival assays cannot supply those missing array-specific controls. Broad pan-PI3K inhibition cannot identify a responsible isoform or exclude off-target and viability-related effects without orthogonal assays. WGCNA was a deterministic secondary analysis after inspection of earlier gene-level results. Part A and Part B share one accession, so their agreement is within-study support rather than external replication. Transcript abundance alone does not establish protein activity, degranulation, tissue injury or therapeutic benefit. No new functional neutrophil experiments were performed. Source-study assays [17] do not directly validate these programme or module scores.

The next decisive experiments should test cell type, perturbation and function together. Primary neutrophils and monocytes or macrophages should be compared under a frozen GM-CSF plus broad pan-PI3K design. Direct functional readouts could include granule release, phagocytosis, cell survival and epithelial injury. An independent longitudinal lung or BALF cohort should then test pre-specified neutrophil and monocyte/macrophage endpoints with resolution, VFD or mortality outcomes. Sample size, time points, primary contrasts and multiplicity rules should be fixed before data inspection. Such studies would determine whether the observed transcriptional sensitivity marks a causal injury pathway, a compensatory response or a context-dependent correlate.

## Conclusions

This multi-cohort analysis defines a bounded transcriptional model of ARDS-associated myeloid activation. The strongest evidence is a paired association between ARDS and a neutrophil effector/degranulation programme, together with its GM-CSF inducibility and sensitivity to broad pan-PI3K inhibition in healthy-donor neutrophils ex vivo. A hypothesis-driven route retained three pathway-restricted directional response anchors, whereas a donor-adjusted whole-transcriptome analysis identified a separate GM-CSF-inducible, pan-PI3K-sensitive coexpression module. Their limited gene-level overlap but convergent enrichment for effector and secretion biology supports functional convergence rather than a single shared molecular signature.

External analyses impose clear limits. The frozen green-module projection showed post hoc monocyte/macrophage-biased localization across eligible cellular comparisons; this does not validate a neutrophil-specific mechanism. Longitudinal outcome tests were inconclusive or unsupported, including the eight-patient BALF contrast. The model therefore warrants targeted functional and independent clinical tests before any prognostic or therapeutic claim.

## Supporting information

S1 Fig Boundary cohorts constrain compartment, independence and aetiological interpretation. a, Patient-paired AM and PBM neutrophil-degranulation programme scores in GSE89953. Lines connect samples from the same patient; points are compartment samples; black crossbars denote arithmetic means. The plot contains 23 quality-control-retained pairs. A separate overlap audit of all 26 pairs established that 26 of 26 GSE89953 AM processed arrays were byte-identical to the corresponding GSE116560 Day-1 AM arrays. b, Mean paired AM-minus-PBM differences for the four pre-specified programmes across all 26 pairs. The neutrophil-degranulation programme was lower in AM than PBM (mean difference, −0.0965; two-sided paired Wilcoxon BH-FDR = 1.79 × 10^-7). Apoptotic-cell clearance, inflammatory response and wound healing were higher in AM. c, Strict-quality-control GSE32707 Day-0 whole-blood neutrophil-degranulation scores across ICU control, systemic inflammatory response syndrome, sepsis and sepsis-ARDS analysis units (n = 82). Points are independent analysis units; black crossbars denote group means. All three pre-specified sepsis-ARDS contrasts had BH-FDR ≥ 0.0628. d, GSE207077 BALF neutrophil-degranulation scores in influenza-ARDS (n = 9) and COVID-19-ARDS (n = 19). The COVID-19-minus-influenza difference was 0.00388 (two-sided Wilcoxon P = 0.847; BH-FDR = 0.847). GSE89953 is a compartment audit rather than an independent cohort; GSE32707 is static mixed-cell whole blood; and GSE207077 is a cross-sectional aetiology comparison without healthy controls, outcome or longitudinal sampling. Absence of an aetiology difference does not establish equivalence.
(TIF)

S2 Fig Exploratory monocyte/macrophage wound-healing comparison in GSE240580. Patient-level monocyte/macrophage wound-healing programme scores are shown at baseline (t1) and follow-up (t2), stratified by clinical resolution at t2. Points represent patients, shapes denote aetiology and black crossbars denote group means. At t1, the not-resolved minus resolved contrast was −0.0303 (two-sided exact Wilcoxon P = 0.0357; BH-FDR = 0.286). At t2, the contrast was 0.00487 (P = 1.000; BH-FDR = 1.000). Eight patients contributed paired t1 and t2 samples. The nominal baseline result did not pass multiplicity correction and was absent at follow-up; it is exploratory and does not alter the pre-specified GSE240580 neutrophil-degranulation result.
(TIF)

S3 Fig Genome-wide directional-conjunction and anchor-selection diagnostic in GSE76293. a, Genome-wide directional convergence. Each point is one gene. The x axis shows the paired ARDS-minus-healthy-volunteer effect, the y axis shows GM-CSF induction, and colour denotes the positive reversal magnitude after broad pan-PI3K inhibition. Triangles identify SERPINB1, PNP and TLR2, which were retained only within the detected membership of the pre-specified neutrophil-degranulation programme. b, Genome-wide intersection-union test. Genes are ranked by the maximum of the three pre-specified one-sided P values. The dashed line denotes conjunction BH-FDR = 0.05. No gene passed this threshold; the minimum genome-wide BH-FDR was 0.116. c, Paired Cohen's dz values for SERPINB1, PNP and TLR2 across the three contrasts. d, Frozen decision-rule attrition on a log10(n + 1) display scale. A total of 5,585 genes had positive effects in all three directions, and 251 had three one-sided P values below 0.05. None passed genome-wide conjunction BH-FDR ≤ 0.05 or the Tier-1 strict rule. Three genes passed the Tier-2 pathway-restricted rule. Biological units were 12 matched Part A pairs and 10 paired Part B donors. The three genes are directional anchors, not a genome-wide core module or green-module members.
(TIF)

S4 Fig Directional anchors and the GSE273149 longitudinal boundary. a-c, Paired expression differences for SERPINB1, PNP and TLR2 in GSE76293. a, Twelve matched ARDS-minus-healthy-volunteer neutrophil pairs. b, GM-CSF-minus-DMSO effects in ten healthy donors. c, Positive reversal magnitudes, defined as GM-CSF minus GM-CSF plus broad pan-PI3K inhibitor, in the same donors. Boxes summarize distributions; open circles and error bars denote means and standard errors. These genes passed the frozen rule only within the pre-specified detected neutrophil-degranulation membership and were not genome-wide conjunction-FDR significant. d, Frozen unweighted within-sample percentile-rank score of SERPINB1, PNP and TLR2 across 49 longitudinal GSE273149 PBMC samples. Lines connect samples from the same patient. Four non-COVID-19 ARDS patients provide aetiological context; the frozen analysis uses four COVID-19-ARDS survivors and five non-survivors. e, Patient-specific score-on-day slopes. The non-survivor-minus-survivor difference was 0.00224 score units per day (exact one-sided patient-label permutation P = 0.2143; leave-one-out positive-direction fraction = 1.000). f, Exact null distribution from all 126 allocations of five non-survivor labels among nine patient slopes; the dashed line marks the observed difference. The external result is direction-only/inconclusive, not outcome validation or pulmonary replication.
(TIF)

S5 Fig Extended WGCNA diagnostics and frozen-selection robustness. a, Full soft-threshold diagnostics for the signed donor-adjusted GSE76293 Part B network. Power 8 was the first candidate with signed scale-free-fit R² ≥ 0.80 and was selected deterministically; orange points identify the selected power. b, Complete Part B donor-by-condition design. Each tile represents one sample and displays its RNA integrity number. Ten donors each contributed one sample to six conditions; all 60 samples passed WGCNA sample-quality control. Donor and RNA integrity number were adjusted while the condition design was preserved. c, Gene-membership stability of the primary power-8 green module at adjacent powers. Best-matched modules shared 601 of 716 union genes at power 7 (Jaccard index, 0.839) and 593 of 708 genes at power 9 (Jaccard index, 0.838). Module colours are run-specific, so stability is defined by membership rather than colour name. d, Sequential attrition under the frozen Part B rules. Thirteen non-grey modules were reduced to two after requiring concordant positive effects, paired effect sizes, positive fractions and leave-one-donor-out directions; conjunction BH-FDR ≤ 0.05 retained green alone. The network included the 10,827 genes in the top half of donor-adjusted median absolute deviation among 21,653 input genes. This figure provides technical robustness, not independent validation.
(TIF)

S6 Fig Frozen green-module members and Part B hubs. Complete display of the 627 genes in the retained green module. The x axis is the smaller of the two mean Part B gene effects, GM-CSF induction and broad pan-PI3K reversal; the y axis is oriented module membership (kME). Open points denote module members and orange points denote the 86 frozen hubs. The horizontal dashed line marks kME = 0.70 and the vertical line marks zero minimum effect. Hubs were defined using Part B only by kME ≥ 0.70, positive mean effects in both contrasts and positive donor fractions of at least 0.70 in both contrasts. The five highest-kME hubs are labelled. Neither Part A nor external-cohort results were used to alter membership. The display records membership and the frozen hub rule; it does not imply individual-gene significance or therapeutic validation.
(TIF)

S7 Fig Full frozen top-three nonredundant ORA profile. Full frozen over-representation analysis profile for the complete 627-gene green module and the 86 frozen hubs across Gene Ontology Biological Process, Hallmark and Reactome libraries. The display contains the top three redundancy-pruned significant terms for each eligible query-by-library combination, or all retained terms when fewer than three were available, yielding 15 terms. Point position denotes fold enrichment on a logarithmic scale, point area denotes the overlap count and colour denotes -log10(BH-FDR). No pathway was manually selected for display; complete pruned results remain in the source data. The profile supports bounded functional convergence but does not establish identical gene membership, GM-CSF-specific enrichment, canonical PI3K/AKT enrichment, causal validation or an outcome-predictive signature.
(TIF)

S8 Fig Fixed-loading green-module external projection. a, GSE151263 blood-cell localization. Frozen weighted rank scores are shown for eligible patient-level cell-state pseudobulks; points denote patients and boxes summarize distributions. The three-state myeloid Friedman comparison uses seven patients. The monocyte-high ordering is post hoc and hypothesis-generating; no ARDS-versus-sepsis inference is made because of the seven-patient design and sex confounding. b, GSE240580 BALF localization. Baseline broad-cell pseudobulk scores are shown at left; paired monocyte/macrophage and neutrophil scores from eight patients are shown at right. The mean neutrophil-minus-monocyte/macrophage difference was −0.06554 (one-sided exact Wilcoxon P = 1.000). c, GSE240580 paired BALF course. The frozen non-remission-minus-remission change contrast was 0.04100 (exact one-sided permutation P = 0.1786; leave-one-out positive-direction fraction = 1.000). d, GSE273149 PBMC trajectories and patient slopes by survival status. The non-survivor-minus-survivor slope contrast was −0.0002079 per day (exact one-sided P = 0.5714; leave-one-out positive-direction fraction = 0.333). e, Platform-limited GSE116560 sensitivity. Weighted-score time-by-standardized-ventilator-free-day interaction coefficients and 95% confidence intervals are shown across all-sample, age/sex-adjusted and quality-control-retained tracks. Only 60.6% of genes and 63.6% of loading mass were mapped, below the frozen 70% coverage gate. Scores use the 627 frozen green-module members and original Part B PC1 loadings with no external refitting. Absolute scores are not compared across cohorts.
(TIF)

S1 Data Individual figure values, network records and Fig 8d provenance. (ZIP). (ZIP)
