# Supplementary material for: Paired neutrophil association and ex vivo perturbation sensitivity define a bounded myeloid activation model in ARDS
Source: PLoS One. 2026 Nov 12;21(11):e0362747. doi: 10.1371/journal.pone.0362747 (PMC13759100; doi:10.1371/journal.pone.0362747)
Supplement: Peer review history

# Peer review: Decision Letter 0

**DOI:** 10.1371/journal.pone.0362747.r001

**Authors:** Tomasz Kaminski

6 Oct 2026

Dear Dr. Zhang,

Thank you for submitting your manuscript to PLOS One. After careful consideration, we feel that it has merit but does not fully meet PLOS One’s publication criteria as it currently stands. Therefore, we invite you to submit a revised version of the manuscript that addresses the points raised during the review process.

Please submit your revised manuscript by Dec 05 2026 11:59PM. If you will need more time than this to complete your revisions, please reply to this message or contact the journal office at plosone@plos.org. When you're ready to submit your revision, log on to https://www.editorialmanager.com/pone/ and select the 'Submissions Needing Revision' folder to locate your manuscript file.

- A letter that responds to each point raised by the academic editor and reviewer(s). You should upload this letter as a separate file labeled 'Response to Reviewers'.
- A marked-up copy of your manuscript that highlights changes made to the original version. You should upload this as a separate file labeled 'Revised Manuscript with Track Changes'.
- An unmarked version of your revised paper without tracked changes. You should upload this as a separate file labeled 'Manuscript'.

If you would like to make changes to your financial disclosure, please include your updated statement in your cover letter. Guidelines for resubmitting your figure files are available below the reviewer comments at the end of this letter.

If applicable, we recommend that you deposit your laboratory protocols in protocols.io to enhance the reproducibility of your results. Protocols.io assigns your protocol its own identifier (DOI) so that it can be cited independently in the future. For instructions see: https://journals.plos.org/plosone/s/submission-guidelines#loc-laboratory-protocols. Additionally, PLOS One offers an option for publishing peer-reviewed Lab Protocol articles, which describe protocols hosted on protocols.io. Read more information on sharing protocols at https://plos.org/protocols?utm_medium=editorial-email&utm_source=authorletters&utm_campaign=protocols.

As the corresponding author, your ORCID iD is verified in the submission system and will appear in the published article. PLOS supports the use of ORCID, and we encourage all coauthors to register for an ORCID iD and use it as well. Please encourage your coauthors to verify their ORCID iD within the submission system before final acceptance, as unverified ORCID iDs will not appear in the published article. *Only*  the individual author can complete the verification step; PLOS staff *cannot*  verify ORCID iDs on behalf of authors.

We look forward to receiving your revised manuscript.

Kind regards,

Tomasz W. Kaminski

Academic Editor

PLOS One

Journal Requirements:

When submitting your revision, we need you to address these additional requirements.

1. Please ensure that your manuscript meets PLOS ONE's style requirements, including those for file naming. The PLOS ONE style templates can be found at

https://journals.plos.org/plosone/s/file?id=wjVg/PLOSOne_formatting_sample_main_body.pdf and

https://journals.plos.org/plosone/s/file?id=ba62/PLOSOne_formatting_sample_title_authors_affiliations.pdf

2. Please amend the manuscript submission data (via Edit Submission) to include author LinGang Zhang.

3. Please amend your authorship list in your manuscript file to include author LingGang Zhang.

4. Please upload a copy of Figure 6, to which you refer in your text on page 27. If the figure is no longer to be included as part of the submission please remove all reference to it within the text.

5. If the reviewer comments include a recommendation to cite specific previously published works, please review and evaluate these publications to determine whether they are relevant and should be cited. There is no requirement to cite these works unless the editor has indicated otherwise.

Additional Editor Comments:

Dear Authors,

The manuscript presents a rigorous and thoughtfully structured transcriptomic analysis of neutrophil/myeloid programs in ARDS, with particularly strong statistical reporting and transparent handling of limitations. However, several important issues require substantial revision before the manuscript can be considered further.

The main concerns relate to the small and heterogeneous datasets, the heavy dependence of the mechanistic interpretation on GSE76293, and the lack of functional validation of the proposed neutrophil effector program. The authors should clearly distinguish discovery, within-dataset replication, external validation, and exploratory analyses; reconsider the interpretation of the sex-confounded GSE151263 comparison; clarify the cell-type dependence of the identified transcriptional programs; and provide complete methodological details regarding the pan-PI3K inhibitor, including compound, concentration, vehicle, rationale, and viability assessment. In addition, the mechanistic conclusions would be considerably strengthened by functional neutrophil readouts such as degranulation, MPO release, CD11b expression, ROS production, NETosis, or related assays, and by a more careful discussion of PI3K isoform specificity and the limitations of using healthy donor rather than ARDS neutrophils.

Overall, the study is potentially valuable, but the current conclusions should be more tightly aligned with the strength of the available evidence.

Best regards,

Tomasz W Kaminski

[Note: HTML markup is below. Please do not edit.]

Reviewers' comments:

Reviewer's Responses to Questions

**Comments to the Author**

1. Is the manuscript technically sound, and do the data support the conclusions?

Reviewer #1: Yes

Reviewer #2: Partly

**********

2. Has the statistical analysis been performed appropriately and rigorously?

Reviewer #1: Yes

Reviewer #2: I Don't Know

**********

3. Have the authors made all data underlying the findings in their manuscript fully available?

The PLOS Data policy

Reviewer #1: Yes

Reviewer #2: No

**********

4. Is the manuscript presented in an intelligible fashion and written in standard English?

Reviewer #1: Yes

Reviewer #2: Yes

**********

Reviewer #1: This manuscript primarily focused on neutrophil effector and/or degranulation programs and their relationship to GM-CSF signaling and PI3K pathway modulation. This manuscript is enriched with methodological rigor with the inferential basis and all the transparent handling of the data. The use of statistical framework is exceptional to present the data comprehensively. The hypothesis and data driven approach is showing interesting, limited gene overlap and support the biological relevance.

Major insights:

The manuscripts central claim could be enhanced by including some mechanistic studies like degranulation, NETosis, phagocytosis, respiratory burst, or other functional neutrophil outputs as the transcriptional changes may not reflect to altered effector function. Although the author acknowledged the limitation of not having the neutrophils from ARDS patients, but having those neutrophils could have substantially uplift the inference. Some insights on PI3K isoforms will be helpful and relevant as they have distinct roles in neutrophil biology. Also, small sample sizes are a limitation, but the data outcome can be interpreted as support not evidence.

The GSE151263 PBMC analysis comparing ARDS to sepsis suffers from complete confounding with sex—all 3 ARDS patients have female expression proxies, while 3 of 4 sepsis patients have male proxies. This is a fatal flaw for any disease comparison from this cohort. The authors correctly report this, but the analysis still appears in the main results as a "negative" finding.

The pre-specified programs (particularly neutrophil degranulation) are heavily enriched for neutrophil biology, yet:

They localize to monocytes/macrophages in PBMC (Fig. 2) The green module projection shows monocyte/macrophage bias (Fig. 8b). The core disease association is in purified neutrophils (GSE76293). This raises questions about whether the same transcriptional program has different meaning in different cell types. The authors note this but could more explicitly explore what this means for the biological interpretation.

Minor insights:

Abstract line 33-34: "paired BALF endpoint did not reproduce the proposed non-resolution direction"—this is too strongly stated as a "negative" finding given n=8.

Methods section: The description of the WGCNA approach is dense—consider adding a schematic or more accessible summary.

Figure 8d: The integrated evidence model is helpful but could be more clearly labeled in terms of what is supported vs. unsupported.

Reviewer #2: Manuscript ID: PONE-D-26-43534

Manuscript Title: Paired neutrophil association and ex vivo perturbation sensitivity define a bounded myeloid activation model in ARDS

In this study, LingGang Zhang and colleagues investigated how myeloid transcriptional programmes relate across blood and lung compartments in acute respiratory distress syndrome (ARDS). The authors found that ARDS-associated myeloid/neutrophil effector programme with paired disease association and ex vivo GM-CSF/pan-PI3K sensitivity. They support mechanistic prioritization of compartment-dependent myeloid biology, but not a prognostic signature, a causal programme-to-outcome mechanism like PI3K-directed therapeutic efficacy. However, there are several important issues that should be addressed before consideration.

Comments:

1. The main concern in this article is that the important findings are derived from small, heterogeneous public datasets, while the mechanistic interpretation depends on one accession i.e. GSE76293.

2. It would be good if the authors more clearly distinguish b/w discovery, within-accession replication, external validation, and exploratory contextual analyses.

3. The use of healthy donor neutrophils for mechanistic conclusion is a major limitation. The experiment uses healthy donor neutrophils exposed ex vivo to GM-CSF and broad pan-PI3K inhibitor. This determines the transcriptional responsiveness, but it does not establish that the same pathway works in ARDS patient neutrophils in vivo.

4. Broad pan-PI3K inhibition cannot prove PI3K-dependent mechanism. A pan PI3K inhibitor can potentially have isoform-independent effects, off-target effects, effects on cell viability, and concentration-dependent nonspecific effects. Some key analyses involve very less numbers of patients. Example: GSE151263 have 3 ARDS vs 4 sepsis patients. GSE240580 has 8 paired patients and GSE273149 have 9 COVID-19 ARDS patients for survival analysis.

5. The authors should provide more information about the used inhibitor including compound, its concentration, vehicle concentration, evidence for the selected concentration and whether neutrophil viability was evaluated.

6. There is no functional support for neutrophil degranulation processes like MPO release, surface CD11b changes, ROS production and other if any.

7. ARDS patients receive multiple therapies that can affect the neutrophil transcriptional states and its functions. The authors should determine whether related treatment information is available and if so, it should be incorporated for clear interpretation.

8. The manuscript repeatedly states that the data does not establish causality, its prognosis, PI3K isoform specificity, and therapeutic efficacy. These limitations are important. However, repetition can be reduced if possible.

**********

what does this mean?). If published, this will include your full peer review and any attached files.

If you choose “no”, your identity will remain anonymous but your review may still be made public.

**Do you want your identity to be public for this peer review?**  For information about this choice, including consent withdrawal, please see our Privacy Policy

Reviewer #1: No

Reviewer #2: No

**********

[NOTE: If reviewer comments were submitted as an attachment file, they will be attached to this email and accessible via the submission site. Please log into your account, locate the manuscript record, and check for the action link "View Attachments". If this link does not appear, there are no attachment files.]

To ensure your figures meet our technical requirements, please review our figure guidelines: https://journals.plos.org/plosone/s/figures

You may also use PLOS’s free figure tool, NAAS, to help you prepare publication quality figures: https://journals.plos.org/plosone/s/figures#loc-tools-for-figure-preparation.

NAAS will assess whether your figures meet our technical requirements by comparing each figure against our figure specifications.

# Author comment: Author response to Decision Letter 1

**DOI:** 10.1371/journal.pone.0362747.r002

14 Oct 2026

Response to the Editor and Reviewers

Manuscript: PONE-D-26-43534, Paired neutrophil association and ex vivo perturbation sensitivity define a bounded myeloid activation model in ARDS

Corresponding author: LinGang Zhang

Dear Editor and Reviewers,

Thank you for the careful assessment. We revised the manuscript to separate paired disease association, healthy-donor ex vivo perturbation, within-accession projection, and external contextual analyses. We removed disease inference from the sex-confounded GSE151263 comparison, clarified the biological meaning of scores across cell types, moderated the small BALF finding, and restricted the inhibitor interpretation to transcriptional sensitivity. We audited treatment metadata and the source data underlying the figures. The responses below identify the remaining information we could not verify rather than assigning results from a different experiment to the 6-hour arrays.

Journal requirements

JR1. Manuscript format and file naming

Original journal requirement: 1. Please ensure that your manuscript meets PLOS ONE's style requirements, including those for file naming. The PLOS ONE style templates can be found at https://journals.plos.org/plosone/s/file?id=wjVg/PLOSOne_formatting_sample_main_body.pdf and https://journals.plos.org/plosone/s/file?id=ba62/PLOSOne_formatting_sample_title_authors_affiliations.pdf

Response: We prepared a clean revised manuscript and a separate Word Track Changes copy against the original submission. The locally rendered clean manuscript has 51 pages, continuous line numbers and a 295-word abstract. We corrected title-page affiliation punctuation and an Acknowledgments style error. The eight main figures are available as separate, single-page 300-dpi TIFFs, and S1–S8 are available as separate TIFF Supporting Information files. The corresponding captions and citations are in the manuscript; the supplementary item labels now match their S1_Fig.tif–S8_Fig.tif filenames.

Manuscript locations: p. 1, line 5; p. 41, line 926; pp. 46–51, lines 1031–1148.

JR2–JR3. Author name in the submission data and manuscript

Original journal requirements: 2. Please amend the manuscript submission data (via Edit Submission) to include author LinGang Zhang. 3. Please amend your authorship list in your manuscript file to include author LingGang Zhang.

Response: The correct author name is LinGang Zhang. The corresponding author has corrected the PLOS ONE personal profile. The manuscript author line, correspondence information and author contributions use LinGang Zhang. We understand “LingGang” in the manuscript request to be a spelling discrepancy, and have retained the author-confirmed spelling.

Manuscript locations: p. 1, line 5; p. 41, line 920.

JR4. Figure 6

Original journal requirement: 4. Please upload a copy of Figure 6, to which you refer in your text on page 27. If the figure is no longer to be included as part of the submission please remove all reference to it within the text.

Response: Fig. 6 remains cited and captioned in the revised manuscript. We prepared Figure6.tif as a separate, single-page, 300-dpi main-figure file for the revision.

Manuscript locations: p. 28, line 629.

JR5. Suggested citations

Original journal requirement: 5. If the reviewer comments include a recommendation to cite specific previously published works, please review and evaluate these publications to determine whether they are relevant and should be cited. There is no requirement to cite these works unless the editor has indicated otherwise.

Response: The supplied reviewer comments contain no specific publication recommendation. We found no requested citation to add under this item.

Manuscript locations: No manuscript change was required for this item.

Editor: scope, evidence boundaries and methodological details

Original editor comment: The main concerns relate to the small and heterogeneous datasets, the heavy dependence of the mechanistic interpretation on GSE76293, and the lack of functional validation of the proposed neutrophil effector program. The authors should clearly distinguish discovery, within-dataset replication, external validation, and exploratory analyses; reconsider the interpretation of the sex-confounded GSE151263 comparison; clarify the cell-type dependence of the identified transcriptional programs; and provide complete methodological details regarding the pan-PI3K inhibitor, including compound, concentration, vehicle, rationale, and viability assessment. In addition, the mechanistic conclusions would be considerably strengthened by functional neutrophil readouts such as degranulation, MPO release, CD11b expression, ROS production, NETosis, or related assays, and by a more careful discussion of PI3K isoform specificity and the limitations of using healthy donor rather than ARDS neutrophils. Overall, the study is potentially valuable, but the current conclusions should be more tightly aligned with the strength of the available evidence.

Response: We clarified the Fig. 1 legend and the Results framework to identify each cohort's compartment and evidentiary role. The author selected the corrected Fig. 1 raster candidate, which labels the small BALF endpoint inconclusive; it is staged locally with the other figures. GSE76293 Part A supplies paired ARDS–healthy-volunteer purified-neutrophil association; Part B supplies a separate healthy-donor ex vivo perturbation and network selection. The Part A projection of the Part B module is within-accession, direction-only evidence (one-sided BH-FDR 0.0659), not independent external validation. External fixed-loading projections and the small longitudinal outcome tests are contextual or exploratory according to their coverage and design; the eligible outcome tests did not validate a prognostic signal. GSE89953 overlaps GSE116560 and is not counted as independent replication. The revised Abstract, Results, Discussion and Fig. 8d reflect this hierarchy.

GSE151263 is now descriptive PBMC cell-state localization only. All three ARDS patients had female expression-inferred sex proxies, whereas three of four sepsis patients had male proxies and one had a female proxy. These are unverified expression proxies, and the severe seven-patient imbalance prevents a credible ARDS–sepsis disease comparison. We removed the nonsignificant disease tests from the main Results and rebuilt Fig. 2d without a disease-group axis or disease-contrast FDR labels; archived tests remain in source data for transparency.

The Methods now identify the 6-hour GSE76293 Part B conditions as recombinant human GM-CSF 1 ng/mL, ZSTK474 10 μM (a pan-class I PI3K inhibitor), and DMSO vehicle. The exact array-specific final DMSO percentage, rationale for choosing 10 μM for these arrays, and condition-matched 6-hour viability could not be verified in the retrieved source records. The source study's 0.1% DMSO and Annexin V/propidium iodide results belong to a separate 20-hour survival assay and are not presented as array controls. We now describe ZSTK474-sensitive ex vivo transcriptional change, without assigning a PI3K isoform, excluding off-target or viability effects, or claiming therapeutic efficacy. No new MPO-release, CD11b, ROS, NETosis or other functional assay was performed. The Discussion specifies that programme scores do not demonstrate granule release or the same mechanism in ARDS patient cells.

We agree that the overall interpretation should match these limits. The revised Conclusions emphasize a bounded association and perturbation-sensitivity model, with clinical and causal claims unsupported.

Manuscript locations: p. 6, line 115; p. 11, line 235; p. 19, line 409; p. 40, line 885.

Reviewer 1

R1.1. Functional neutrophil outputs, patient cells, PI3K isoforms and small samples

Original reviewer comment: The manuscripts central claim could be enhanced by including some mechanistic studies like degranulation, NETosis, phagocytosis, respiratory burst, or other functional neutrophil outputs as the transcriptional changes may not reflect to altered effector function. Although the author acknowledged the limitation of not having the neutrophils from ARDS patients, but having those neutrophils could have substantially uplift the inference. Some insights on PI3K isoforms will be helpful and relevant as they have distinct roles in neutrophil biology. Also, small sample sizes are a limitation, but the data outcome can be interpreted as support not evidence.

Response: We agree. No new functional or patient-neutrophil experiment was performed. The revised Discussion distinguishes transcriptomic programme scores from granule release, NET formation, phagocytosis and respiratory burst, and specifies that the source study's functional assays do not directly validate the scores calculated in this secondary analysis. ZSTK474 is a broad inhibitor: the data do not identify the responsible PI3K isoform or establish viability-independent, in vivo PI3K dependence. We discuss the separate healthy-donor versus ARDS-neutrophil survival observations in the source study as a reason for caution. Sample sizes and uncertainties remain explicit: 12 disease pairs, ten perturbation donors, eight paired BALF patients, and nine COVID-19 ARDS patients in the external survival analysis. The revised wording uses association and ex vivo sensitivity rather than causal or therapeutic language.

Manuscript locations: p. 11, line 235; p. 40, lines 885 and 896.

R1.2. Sex-confounded GSE151263 comparison

Original reviewer comment: The GSE151263 PBMC analysis comparing ARDS to sepsis suffers from complete confounding with sex—all 3 ARDS patients have female expression proxies, while 3 of 4 sepsis patients have male proxies. This is a fatal flaw for any disease comparison from this cohort. The authors correctly report this, but the analysis still appears in the main results as a "negative" finding.

Response: We agree. The three ARDS patients had female expression-inferred proxies; three of four sepsis patients had male proxies and one had a female proxy. Demographic sex was not independently verified. We removed the nonsignificant ARDS–sepsis tests from the main Results and no longer interpret them as evidence for either presence or absence of a disease effect. Revised Fig. 2d pools patient-level scores within cell states, with no disease-group axis or contrast FDR labels. The Methods note that historical tests remain in the source tables solely for transparency. The Abstract, Results, Discussion, Fig. 2 legend and Fig. 8d now call this a descriptive PBMC localization cohort.

Manuscript locations: p. 8, line 161; p. 21, lines 462 and 473; p. 39, line 870.

R1.3. Cell-type meaning of programmes and green-module projection

Original reviewer comment: The pre-specified programs (particularly neutrophil degranulation) are heavily enriched for neutrophil biology, yet: They localize to monocytes/macrophages in PBMC (Fig. 2) The green module projection shows monocyte/macrophage bias (Fig. 8b). The core disease association is in purified neutrophils (GSE76293). This raises questions about whether the same transcriptional program has different meaning in different cell types. The authors note this but could more explicitly explore what this means for the biological interpretation.

Response: We expanded the Discussion to distinguish the score's gene-expression definition from cell-specific function. A higher rank-based “neutrophil degranulation” score in a PBMC monocyte does not show neutrophil granule release by that cell, nor an ARDS–sepsis effect in GSE151263. The 627-gene green module is a separate data-driven set selected in healthy-donor neutrophils; its external monocyte/macrophage projection identifies where the fixed expression pattern is observed, not where an identical perturbation mechanism operates. The three hypothesis-driven anchors are not green-module members. We interpret these external patterns as broad, compartment-dependent myeloid context.

Manuscript locations: p. 21, line 462; p. 32, line 712; p. 37, line 830.

R1.4. Eight-patient BALF wording in the Abstract

Original reviewer comment: Abstract line 33-34: "paired BALF endpoint did not reproduce the proposed non-resolution direction"—this is too strongly stated as a "negative" finding given n=8.

Response: We agree. The Abstract now states: “In eight paired BALF patients, the pre-specified contrast was directionally opposite but inconclusive (P = 0.571).” The Results retain the patient-level not-resolved-minus-resolved change contrast (−0.0147; two-sided exact Wilcoxon P = 0.571), while the Discussion and Fig. 3 legend state that the small endpoint-specific test cannot establish absence of the proposed direction in other cohorts. A wording-only Fig. 3 candidate labels the observed estimates as opposite-sign and inconclusive; its six quantitative source/statistics files are unchanged from the locked prior version.

Manuscript locations: p. 2, line 35; p. 23, lines 497 and 507; p. 38, line 848.

R1.5. WGCNA accessibility

Original reviewer comment: Methods section: The description of the WGCNA approach is dense—consider adding a schematic or more accessible summary.

Response: We added a short plain-language sequence immediately before the technical WGCNA methods: adjust donor and RNA-integrity effects while retaining treatment means, group correlated genes, test modules for GM-CSF induction and inhibitor reversal across donors, then freeze the selected module before patient-pair and external projections. The detailed method remains for reproducibility.

Manuscript locations: p. 13, line 281.

R1.6. Fig. 8d supported and unsupported claims

Original reviewer comment: Figure 8d: The integrated evidence model is helpful but could be more clearly labeled in terms of what is supported vs. unsupported.

Response: The revised Fig. 8d labels the retained paired purified-neutrophil association, healthy-donor GM-CSF induction and broad-inhibitor attenuation; it separately labels PBMC localization as “Descriptive myeloid context” with “no ARDS–sepsis inference.” The outcome box says “Longitudinal outcome evidence inconclusive,” notes small differing cohorts, and says there is no validated signal. The final box marks clinical prediction and therapeutic efficacy as “NOT SUPPORTED.” The corresponding legend states that eligible longitudinal tests were inconclusive or not supportive.

Manuscript locations: p. 33, line 722.

Reviewer 2

R2.1. Small heterogeneous public datasets and GSE76293 dependence

Original reviewer comment: 1. The main concern in this article is that the important findings are derived from small, heterogeneous public datasets, while the mechanistic interpretation depends on one accession i.e. GSE76293.

Response: We agree. The revised Abstract, cohort map, Results and Discussion distinguish the strongest paired GSE76293 evidence from smaller context and outcome analyses. Part A and Part B are separate paired experiments within one accession, not independent biological validation. We report effect estimates and sample counts beside the relevant findings, retain nonsupportive results, and do not pool unlike compartments into one effect or present external contextual cohorts as mechanistic replication. The final claim is restricted to purified-neutrophil disease association and healthy-donor ex vivo transcriptional sensitivity.

Manuscript locations: p. 19, line 409; p. 36, line 791; p. 39, line 870.

R2.2. Discovery, within-accession replication, external validation and exploratory context

Original reviewer comment: 2. It would be good if the authors more clearly distinguish b/w discovery, within-accession replication, external validation, and exploratory contextual analyses.

Response: The revised Fig. 1 legend and Results assign each analysis an explicit role; the corrected Fig. 1 raster is staged locally. Hypothesis-driven programme analysis and the deterministic secondary WGCNA route both use GSE76293, so they are complementary but not independent. Part B green-module selection precedes a frozen Part A projection, which remains within-accession and below its support threshold. Frozen external projections are gated by mapped-gene and loading-mass coverage; eligible datasets provide cellular context or small longitudinal tests, whereas GSE116560 is platform-limited. GSE89953 overlaps GSE116560. We do not call these analyses external validation of a prognostic signature or causal mechanism.

Manuscript locations: p. 19, line 421; p. 32, line 712.

R2.3. Healthy-donor neutrophils

Original reviewer comment: 3. The use of healthy donor neutrophils for mechanistic conclusion is a major limitation. The experiment uses healthy donor neutrophils exposed ex vivo to GM-CSF and broad pan-PI3K inhibitor. This determines the transcriptional responsiveness, but it does not establish that the same pathway works in ARDS patient neutrophils in vivo.

Response: We agree. We now state that GSE76293 Part B establishes only healthy-donor ex vivo transcriptional responsiveness. The source study's separate survival assay showed a different response in ARDS blood neutrophils, further limiting extrapolation. Direct disease-cell pathway and functional studies would be required to establish the in vivo mechanism. No such experiment was added in this revision.

Manuscript locations: p. 6, line 115; p. 40, line 885.

R2.4. Pan-PI3K specificity, viability and small cohorts

Original reviewer comment: 4. Broad pan-PI3K inhibition cannot prove PI3K-dependent mechanism. A pan PI3K inhibitor can potentially have isoform-independent effects, off-target effects, effects on cell viability, and concentration-dependent nonspecific effects. Some key analyses involve very less numbers of patients. Example: GSE151263 have 3 ARDS vs 4 sepsis patients. GSE240580 has 8 paired patients and GSE273149 have 9 COVID-19 ARDS patients for survival analysis.

Response: We removed causal “PI3K-dependent” interpretations. ZSTK474-sensitive transcriptional attenuation alone cannot identify a PI3K isoform or rule out off-target, dose or viability effects; matched 6-hour array viability was not verified. We identify the seven-patient sex-confounded GSE151263 cohort as descriptive only, call the eight-patient paired BALF contrast inconclusive, and present the nine-patient COVID-19 ARDS survival projection as exploratory and nonsupportive. These small cohorts do not upgrade the GSE76293 result to a validated clinical outcome finding.

Manuscript locations: p. 11, line 235; p. 39, line 870; p. 40, line 885.

R2.5. Inhibitor compound, dose, vehicle, rationale and viability

Original reviewer comment: 5. The authors should provide more information about the used inhibitor including compound, its concentration, vehicle concentration, evidence for the selected concentration and whether neutrophil viability was evaluated.

Response: The Methods now name ZSTK474 as a pan-class I PI3K inhibitor at 10 μM, combined with recombinant human GM-CSF at 1 ng/mL for 6 hours, and identify DMSO as vehicle. The original paper used 10 μM ZSTK474 in a separate 20-hour apoptosis assay, which gives contextual precedent but is not a verified dose-selection rationale for the arrays. Its reported 0.1% DMSO and Annexin V/propidium iodide measurements likewise cannot be transferred to the 6-hour array conditions. We could not verify the arrays' final DMSO percentage or condition-matched 6-hour viability from the retrieved study and GEO records. The Methods, Fig. 5d legend and Discussion now say this plainly.

Manuscript locations: p. 6, line 115; p. 11, line 235; p. 26, line 577; p. 40, line 885.

R2.6. Direct neutrophil degranulation assays

Original reviewer comment: 6. There is no functional support for neutrophil degranulation processes like MPO release, surface CD11b changes, ROS production and other if any.

Response: We agree. No new functional experiment was performed. We limit “degranulation programme” to a defined transcriptional score and specify that it does not show granule release, CD11b change, ROS production or NETosis. The available source-study functional observations do not validate the programme or green-module scores calculated here. The Discussion identifies direct assays in relevant patient-derived cells as the necessary next test.

Manuscript locations: p. 40, line 896.

R2.7. Patient treatments

Original reviewer comment: 7. ARDS patients receive multiple therapies that can affect the neutrophil transcriptional states and its functions. The authors should determine whether related treatment information is available and if so, it should be incorporated for clear interpretation.

Response: We audited accessible source-study tables and GEO annotations, directly rechecked the GSE240580 steroid row and GSE116560 steroid cells against their supplements, and added a cohort-specific Methods paragraph. GSE240580 reported a daily maximum steroid dose for seven of eight patients; GSE116560 reported systemic steroids at some time for six of 35 and originated in a fish-oil trial, though individual allocation was absent from our GEO annotations. In GSE273149, dexamethasone was reported for all four COVID-19 ARDS survivors and four of five non-survivors, with source-study group-level remdesivir and convalescent-plasma reporting; the four non-COVID ARDS patients had no dexamethasone recorded. GSE76293 Part A GEO records did not include patient medication fields. Treatment timing and comparable dose relative to each transcriptomic sample were incomplete, and in some small cohorts treatment nearly coincided with group membership. We therefore report therapy as plausible heterogeneity and do not fit an unsupported treatment-adjusted model or attribute transcriptional differences solely to disease biology.

Manuscript locations: p. 6, line 130; p. 39, line 870.

R2.8. Repetition of limitations

Original reviewer comment: 8. The manuscript repeatedly states that the data does not establish causality, its prognosis, PI3K isoform specificity, and therapeutic efficacy. These limitations are important. However, repetition can be reduced if possible.

Response: We removed a near-duplicate end-of-Discussion summary and repeated therapeutic/function and longitudinal caveats. The detailed cohort- and inhibitor-specific limitations remain together in the limitations section, while short boundaries stay beside the result to which they apply. These were editorial changes; no estimate, threshold or analysis changed.

Manuscript locations: p. 39, line 870; p. 41, line 906.

R2.9. Underlying data availability (review form answer “No”)

Original review-form question, policy text and answer: 3. Have the authors made all data underlying the findings in their manuscript fully available? The PLOS Data policy requires authors to make all data underlying the findings described in their manuscript fully available without restriction, with rare exception (please refer to the Data Availability Statement in the manuscript PDF file). The data should be provided as part of the manuscript or its supporting information, or deposited to a public repository. For example, in addition to summary statistics, the data points behind means, medians and variance measures should be available. If there are restrictions on publicly sharing data—e.g. participant privacy or use of data from a third party—those must be specified. Reviewer #2: No

Response: We audited all eight main and eight supplementary figures against the public processed figure-data archive, code archive and GEO accessions. The public figure-data archive contains 67 processed source/statistics files, but several panels lacked all individual values behind displayed summaries. We assembled a checksum-verified 12-file S1 Data ZIP with individual Fig. 2b programme-score records; all paired values for panel b of S1 Fig; Fig. 4c–d scores and coefficient tables; donor-level Fig. 6b module-selection values; processed panel c inputs of S5 Fig; and Fig. 8d evidence-node provenance. Reconciliation reproduced the 40 Fig. 2b summaries, four paired mean differences in panel b of S1 Fig, and both mean-effect columns for all 13 Fig. 6b modules. Fig. 1 is a study schematic and Fig. 8d is a provenance-documented evidence model. The S1 Data ZIP has been prepared for inclusion with this revision as PLOS Supporting Information, with a caption at the end of the manuscript. The revised Data Availability statement distinguishes this addendum from the already published Zenodo v1 archive and identifies the GEO accessions for primary data.

Manuscript locations: p. 41, line 926; p. 51, line 1148.

Attachment Submitted filename: PONE-D-26-43534_Response_to_Reviewers.docx

# Peer review: Decision Letter 1

**DOI:** 10.1371/journal.pone.0362747.r003

**Authors:** Tomasz Kaminski

30 Oct 2026

Paired neutrophil association and ex vivo perturbation sensitivity define a bounded myeloid activation model in ARDS

PONE-D-26-43534R1

Dear Dr. Zhang,

We’re pleased to inform you that your manuscript has been judged scientifically suitable for publication and will be formally accepted for publication once it meets all outstanding technical requirements.

Within one week, you’ll receive an e-mail detailing the required amendments. When these have been addressed, you’ll receive a formal acceptance letter and your manuscript will be scheduled for publication.

An invoice will be generated when your article is formally accepted. Please note, if your institution has a publishing partnership with PLOS and your article meets the relevant criteria, all or part of your publication costs will be covered. Please make sure your user information is up-to-date by logging into Editorial Manager at Editorial Manager® and clicking the ‘Update My Information' link at the top of the page. For questions related to billing, please contact billing support.

If your institution or institutions have a press office, please notify them about your upcoming paper to help maximize its impact. If they’ll be preparing press materials, please inform our press team as soon as possible -- no later than 48 hours after receiving the formal acceptance. Your manuscript will remain under strict press embargo until 2 pm Eastern Time on the date of publication. For more information, please contact onepress@plos.org.

Kind regards,

Tomasz W. Kaminski

Academic Editor

PLOS One

Reviewers' comments:

Reviewer's Responses to Questions

**Comments to the Author**

Reviewer #1: All comments have been addressed

Reviewer #2: All comments have been addressed

**********

2. Is the manuscript technically sound, and do the data support the conclusions?

Reviewer #1: Partly

Reviewer #2: Partly

**********

3. Has the statistical analysis been performed appropriately and rigorously?

Reviewer #1: Yes

Reviewer #2: I Don't Know

**********

4. Have the authors made all data underlying the findings in their manuscript fully available?

The PLOS Data policy

Reviewer #1: Yes

Reviewer #2: Yes

**********

5. Is the manuscript presented in an intelligible fashion and written in standard English?

Reviewer #1: Yes

Reviewer #2: Yes

**********

Reviewer #1: Authors are not able to do new functional experiment to support their previous claims but logically modified the discussion based on the data given. The write up is acceptable now according to the discussion. Sex confounded GSE151263 comparison is addressed with the modified results and inference. The other comments and issues are duly addressed.

Reviewer #2: The authors have sufficiently addressed all of my concern and improved the manuscript in the revised version.

**********

what does this mean?). If published, this will include your full peer review and any attached files.

If you choose “no”, your identity will remain anonymous but your review may still be made public.

**Do you want your identity to be public for this peer review?**  For information about this choice, including consent withdrawal, please see our Privacy Policy

Reviewer #1: No

Reviewer #2: No

**********

# Editor report: Acceptance letter

**DOI:** 10.1371/journal.pone.0362747.r004

**Authors:** Tomasz Kaminski

PONE-D-26-43534R1

PLOS One

Dear Dr. Zhang,

I'm pleased to inform you that your manuscript has been deemed suitable for publication in PLOS One. Congratulations! Your manuscript is now being handed over to our production team.

At this stage, our production department will prepare your paper for publication. This includes ensuring the following:

* All references, tables, and figures are properly cited

* All relevant supporting information is included in the manuscript submission,

* There are no issues that prevent the paper from being properly typeset

You will receive further instructions from the production team, including instructions on how to review your proof when it is ready. Please keep in mind that we are working through a large volume of accepted articles, so please give us a few days to review your paper and let you know the next and final steps.

Lastly, if your institution or institutions have a press office, please let them know about your upcoming paper now to help maximize its impact. If they'll be preparing press materials, please inform our press team within the next 48 hours. Your manuscript will remain under strict press embargo until 2 pm Eastern Time on the date of publication. For more information, please contact onepress@plos.org.

You will receive an invoice from PLOS for your publication fee after your manuscript has reached the completed accept phase. If you receive an email requesting payment before acceptance or for any other service, this may be a phishing scheme. Learn how to identify phishing emails and protect your accounts at https://explore.plos.org/phishing.

If we can help with anything else, please email us at customercare@plos.org.

Thank you for submitting your work to PLOS One and supporting open access.

Kind regards,

PLOS One Editorial Office Staff

on behalf of

Dr. Tomasz W. Kaminski

Academic Editor

PLOS One
